# Multimodal analyses of early, untreated systemic sclerosis skin identify a proinflammatory vascular niche of macrophage-fibroblast signaling

**DOI:** 10.1172/jci.insight.198954

**Published:** 2025-12-18

**Authors:** Helen C. Jarnagin, Rezvan Parvizi, Zhiyun Gong, Rosemary Gedert, Xianying Xing, Lam (Alex) C. Tsoi, Rachael Bogle, Madeline J. Morrisson, Laurent Perreard, Patricia A. Pioli, Fred Kolling, Johann E. Gudjonsson, Dinesh Khanna, Michael L. Whitfield

**Affiliations:** 1 Department of Biomedical Data Science, and; 2 Department of Molecular and Systems Biology, Geisel School of Medicine at Dartmouth, Hanover, New Hampshire, USA.; 3 Program in Quantitative Biomedical Science, Dartmouth College, Lebanon, New Hampshire, USA.; 4 Department of Medicine, Division of Rheumatology, University of Michigan, Ann Arbor, Michigan, USA.; 5 Department of Medicine, Division of Dermatology, University of Michigan, Ann Arbor, Michigan, USA.; 6 Department of Microbiology and Immunology, Geisel School of Medicine at Dartmouth, Hanover, New Hampshire, USA.

**Keywords:** Autoimmunity, Dermatology, Fibrosis, Skin, Transcriptomics

## Abstract

Uncovering the early interactions and spatial distribution of dermal fibroblasts and immune cells in treatment-naive patients with diffuse cutaneous systemic sclerosis (SSc) is critical to understanding the earliest events of skin fibrosis. We generated an integrated multiomic dataset of early-stage, treatment-naive diffuse cutaneous SSc skin. Skin biopsies were analyzed by single-nuclei multiome sequencing (snRNA-Seq and snATAC-Seq) and two spatial transcriptomic methods to comprehensively determine molecular changes. We identified an immunomodulatory niche within the papillary, hypodermis, and vascular regions enriched for activated myeloid cells and fibroblasts characterized by expression of genes such as *CXCL12*, *APOE*, and *C7*. Pathway analyses showed significant enrichment of PI3K/AKT/mTOR signaling pathway expression in these cellular niches, driven by profibrotic growth factor signaling networks. Macrophage subclustering showed SSc-specific macrophage activation of IL-6/JAK/STAT signaling and enrichment of oxidative phosphorylation pathways. Ligand-receptor analysis revealed that SSc macrophages secrete PDGF and TGF-β to activate SSc-dominant fibroblast subclusters. Spatial transcriptomic analyses showed monocyte-derived MRC1^+^ macrophages express PDGF near *PDGFR^hi^THY1^hi^* fibroblasts. Multiomic data integration and spatial transcriptomic neighborhood analysis revealed the colocalization of fibroblasts, macrophages, and T cells around the vasculature. These data suggest that interactions between activated immune cells and immunomodulatory fibroblasts around vascular niches are an early event in scleroderma pathogenesis.

## Abstract 2

Multimodal omic approaches of early, untreated systemic sclerosis skin identified a proinflammatory vascular niche characterized by macrophage-fibroblast interactions involving PDGF, TGF-β and PI3K-AKT-mTOR signaling

## Graphical abstract

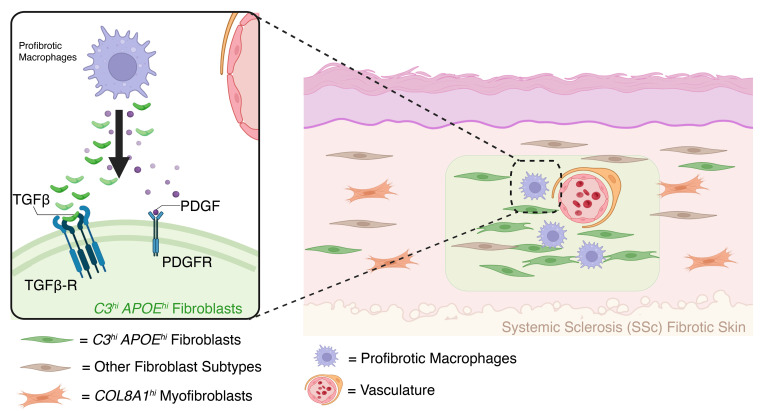

## Introduction

Systemic sclerosis (SSc) is a progressive autoimmune disease characterized by vascular abnormalities, inflammation, and fibrosis of the skin and internal organs (1). The severity and complex clinical diagnosis of the disease make capturing patients with untreated, early progressive disease challenging. Recent single-cell studies highlighted the heterogeneity of fibroblasts and the different fibroblast subpopulations that are implicated in disease pathogenesis (2–5). Understanding the early interactions between fibroblasts and immune cell types within the spatial transcriptomic landscape in treatment-naive patients is critical to identifying and improving therapies for SSc skin fibrosis.

SSc is a heterogeneous disease with variable clinical progression and molecular subtypes, both of which affect the response to therapy. The degree of skin involvement is assessed through a physician-performed evaluation, the modified Rodnan Skin Score (mRSS), and patients can be classified as having either limited cutaneous or diffuse cutaneous SSc depending on the extent of skin involvement (6). Patients often have autoantibodies, which include anti–RNA polymerase antibodies (ARAs; anti-PolI or anti-PolIII), anti–topoisomerase I (Scl-70), or anti-centromere antibodies (6). Gene expression changes in skin and other organs have been used to molecularly stratify patients into groups (inflammatory, fibroproliferative, limited, and normal-like) using bulk gene expression (7, 8). Identifying early disease dynamics remains a critical problem despite these efforts toward patient categorization and classification.

Previous single-cell RNA sequencing (scRNA-Seq) studies sought to understand the individual cell contributions of SSc-related skin fibrosis. This includes dissecting the heterogeneity of fibroblasts, assessing endothelial cell injury, monitoring immune cell contributions, observing alterations based on autoantibody subtype and disease stage, observing changes during drug treatment, and spatial transcriptomic assessment of fibrotic skin (2–5, 9–13). However, these studies were limited to scRNA-Seq and did not include sample-matched ATAC-Seq or spatial transcriptomics to contextualize the results. These studies also included patient samples on background therapy, which may confound results. Here, we sought to comprehensively characterize the cellular and molecular events contributing to SSc skin fibrosis using skin biopsies from a vertically integrated multiomic cohort of treatment-naive patients early in the disease course.

Skin fibrosis and thickening are due to the increased deposition of extracellular matrix (ECM) components by activated fibroblasts, specifically myofibroblasts. Myofibroblasts are mesenchymal cells that contribute to increased skin tightness through excess ECM production. TGF-β activity is the most well-documented source of myofibroblast transformation (14). PDGF, EGF, and FGF signaling are also implicated in multiple types of fibrosis and wound healing (15–19). Nintedanib, a PDGF receptor inhibitor, is one of the few FDA-approved therapies for SSc-associated interstitial lung disease (1). Although nintedanib improves lung function in interstitial lung disease and slows overall disease progression, it did not result in a significant decrease in the mRSS in SSc clinical trials (1). Preclinical studies demonstrated the efficacy of nintedanib in activated dermal fibroblasts, indicating the importance of developing more effective and targeted approaches for the inhibition of a PDGF-inducible pathway (17). Other preclinical studies determined that macrophage-associated PDGF signaling contributes to fibrosis by activating the PI3K/AKT signaling cascade (20). The PI3K/AKT pathway is strongly enriched in an integrated multicohort analysis of gene expression from SSc skin samples, underscoring its importance in SSc pathology (19).

In this study, we generated a vertically integrated dataset on treatment-naive patients with diffuse cutaneous SSc skin that includes single-nuclei multiome (snMultiome) sequencing and paired spatial transcriptomic analyses to comprehensively describe the molecular changes in these individuals. Previous work emphasized the importance of *SFRP2^hi^* fibroblasts as potential progenitors for profibrotic fibroblasts; we were able to demonstrate similar relevance of this population while gaining more insight into the broader population-level dynamics of fibroblasts in patients in the early disease stage (2). Given the well-established immune/fibrotic axis in SSc, we sought to understand the dynamics between macrophages and fibroblasts to identify critical pathways driving the disease. Finally, we demonstrated that the PI3K/AKT pathway relies on PDGF signaling in combination with TGF-β and its downstream effectors to drive early SSc skin fibrosis progression. Spatial transcriptomic analyses identified macrophage interactions with specific immunomodulatory fibroblasts near vascular niches in SSc skin biopsies.

## Results

### snMultiome and spatial transcriptomic sequencing demonstrate relevant cell types and spatial enrichment in SSc skin samples.

**Figure 1:**
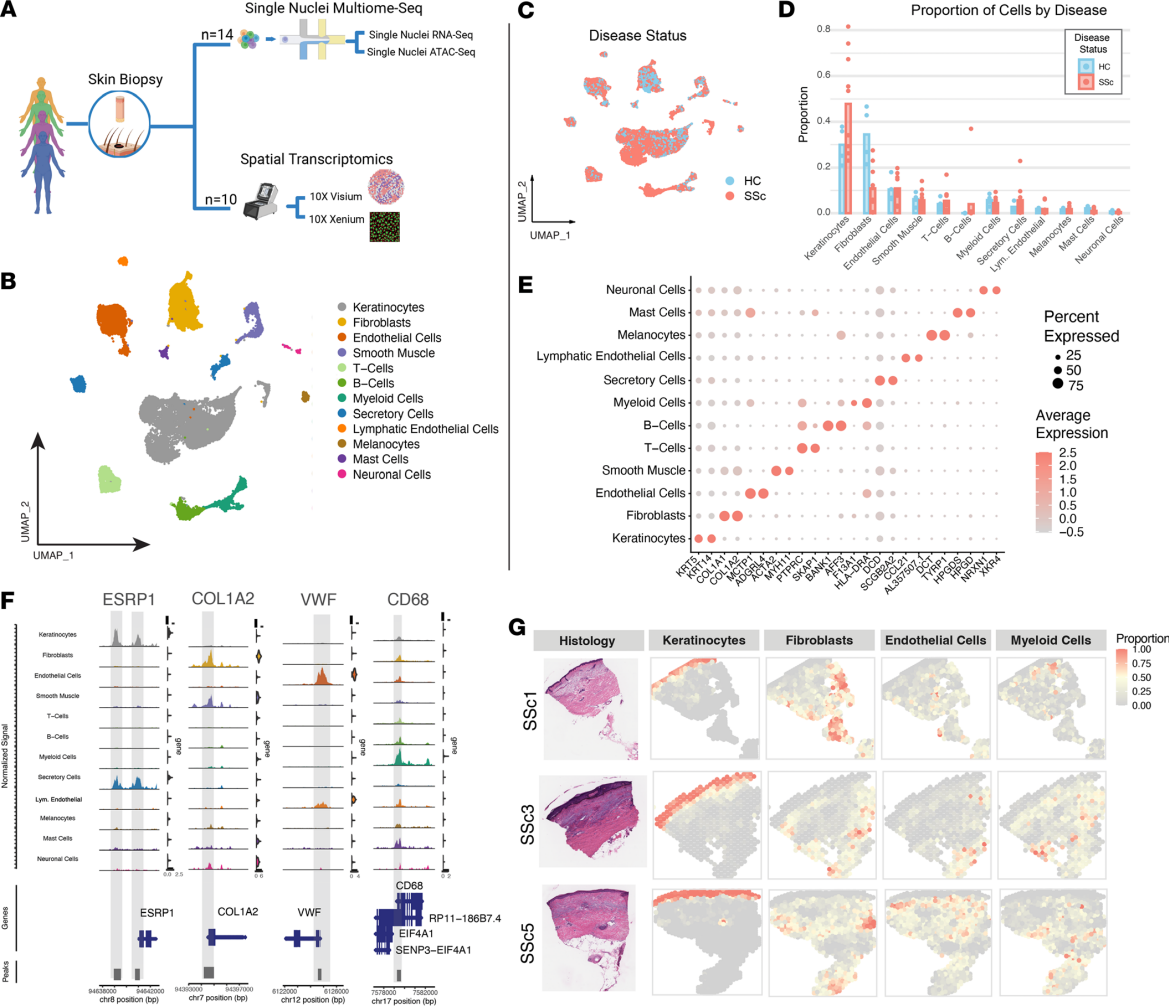
Complete snMultiome clustering and spatial transcriptomics analysis reveal cell-type distribution in SSc skin. (**A**) Experimental schematic outlining sequencing technologies used for each skin biopsy. (BioRender). (**B**) Labeled uniform manifold approximation and projection (UMAP) depicting multimodal clustering of all nuclei harvested from 10 SSc and 4 HC skin samples. (**C**) UMAP demonstrates the broad and even distribution of SSc and HC nuclei. (**D**) Proportional difference of each cell type per patient by disease status. Minor differences in the abundance of cells between the 2 conditions after quality-control filtering (2-tailed *t* test). (**E**) Two of the highest differentially expressed transcripts in each cell type (Wilcoxon’s rank-sum test). (**F**) Significantly differentially accessible (DA) peaks in the ATAC-Seq data near ESPR1 (DA in keratinocytes, *P* = 0), COL1A2 (DA in fibroblasts, *P* = 5.87 × 10^–20^), VWF (DA in endothelial cells, *P* = 2.22 × 10^–251^), and CD68 (DA in myeloid cells, *P* = 4.97 × 10^–54^) (Wilcoxon’s rank-sum test). (**G**) CARD-based deconvolution shows the estimated proportion of major cell types in 3 representative SSc skin sections.

We generated single-nuclei (sn) suspensions and performed snATAC-Seq and snRNA-Seq on 10 SSc skin samples, and 4 age- and sex-matched healthy controls (HCs) (Figure 1A and Table 1). After quality-control filtering, we obtained a dataset of 34,525 nuclei with an average of 2,553 unique genes and 6,895 total transcripts per nucleus. We identified 12 major cell types across the samples from 20 original clusters (Figure 1, B–D, Supplemental Figure 1, A–C, and Supplemental Tables 1 and 2; supplemental material available online with this article; https://doi.org/10.1172/jci.insight.198954DS1). Highly variant genes and ATAC peaks clustered the nuclei on a uniform manifold approximation and projection (UMAP) plot (Figure 1B). Proportional to total nuclei captured, there were slightly more keratinocyte, B cell, T cell, and secretory cell populations in SSc compared with HC samples, but differences were noted (Figure 1D and Supplemental Table 3). These can largely be attributed to sampling effects, which are not necessarily disease or biologically relevant (Figure 1D). Differentially expressed genes and accessible ATAC peaks near canonical genes support canonical cell type assignments as reported by previous scRNA-Seq and bulk transcriptomic studies (Figure 1, E and F) (2, 4). These cell types include, in order of abundance, keratinocytes, fibroblasts, endothelial cells, smooth muscle cells, T cells, B cells, myeloid cells, secretory cells, lymphatic endothelial cells, melanocytes, mast cells, and neuronal cells (Figure 1E).

**Figure 2:**
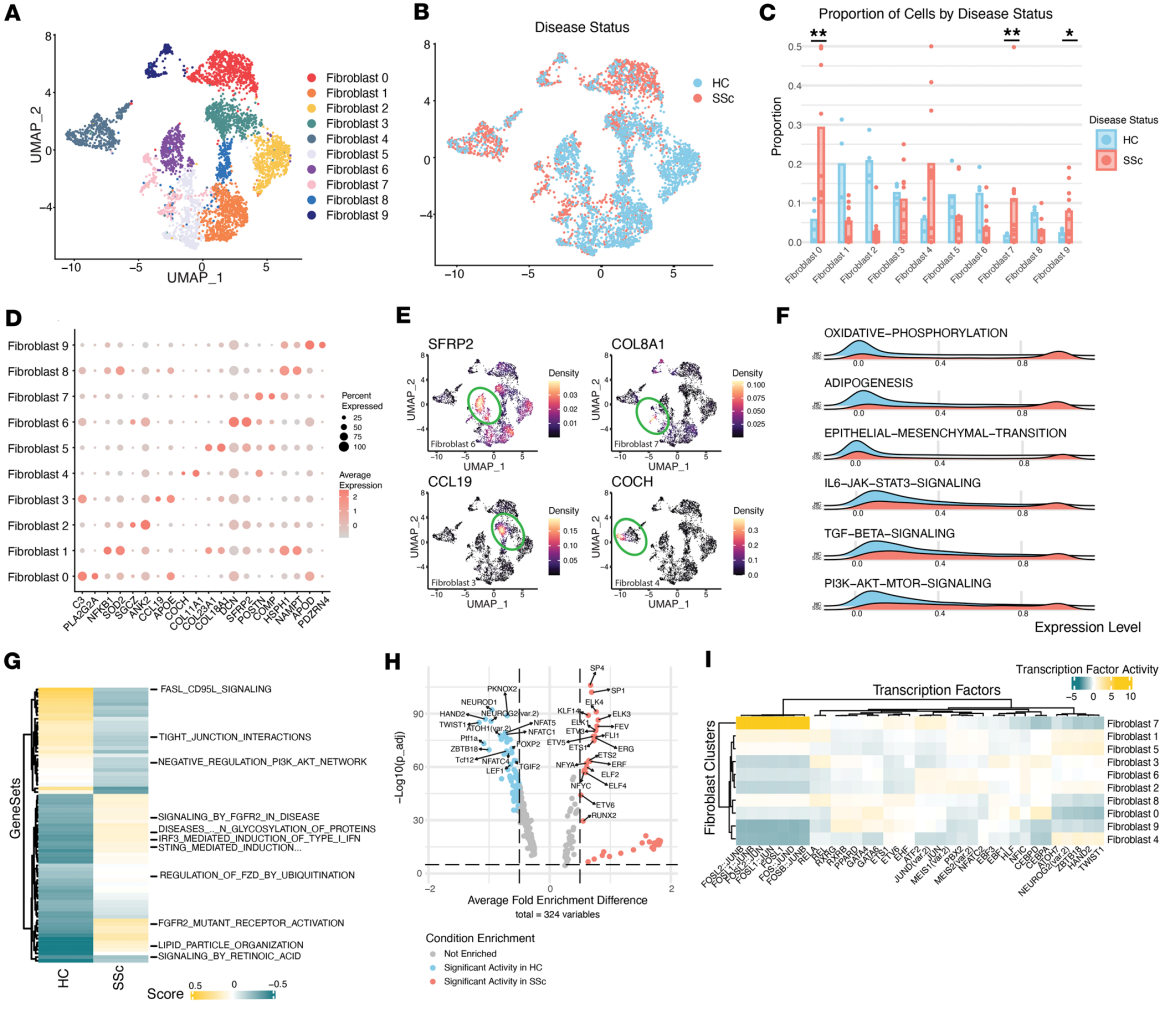
SSc fibroblasts demonstrate major transcriptomic activation in growth factor response and downstream mediators. (**A**) Multimodal UMAP clustering identifies 10 distinct fibroblast subclusters. (**B**) UMAP demonstrating the broad distribution of SSc and HC nuclei in subsets of fibroblasts. (**C**) The proportion of each fibroblast cluster per patient in SSc and HC samples. Fibroblast clusters of 0, 7, and 9 are significantly enriched in SSc skin (2-tailed *t* test: **adjusted *P* value ≤ 0.01, *adjusted *P* value ≤ 0.05). (**D**) Two of the most differentially expressed RNA transcripts from each fibroblast subset. (**E**) UMAP with gene expression density for *SFRP2*, *COLA81*, *CCL19*, and *COCH* enriched across fibroblast subclusters for fibroblasts 6, 7, 2, and 4, respectively. (**F**) Variance-adjusted Mahalanobis (VAM) analysis reveals a global and significant enrichment of specific hallmark pathways, including epithelial-mesenchymal transition, TGF-β signaling, and PI3K Hallmark pathway in SSc compared with HC fibroblasts (Wilcoxon’s rank-sum test, all adjusted *P* ≤ 0.01). (**G**) GSVA of the pseudo-bulked fibroblasts shows significant differential variation in ECM interactions and FGF response and signaling in the SSc samples (Wilcoxon’s rank-sum test, all *P* values ≤ 0.05). (**H**) Volcano plot shows the inferred transcription activity in all SSc or HC fibroblasts (adjusted *P* values by Wilcoxon’s rank-sum test). (**I**) The top most enriched transcription factors by activity for each fibroblast subcluster for both SSc and HC samples.

**Figure 3:**
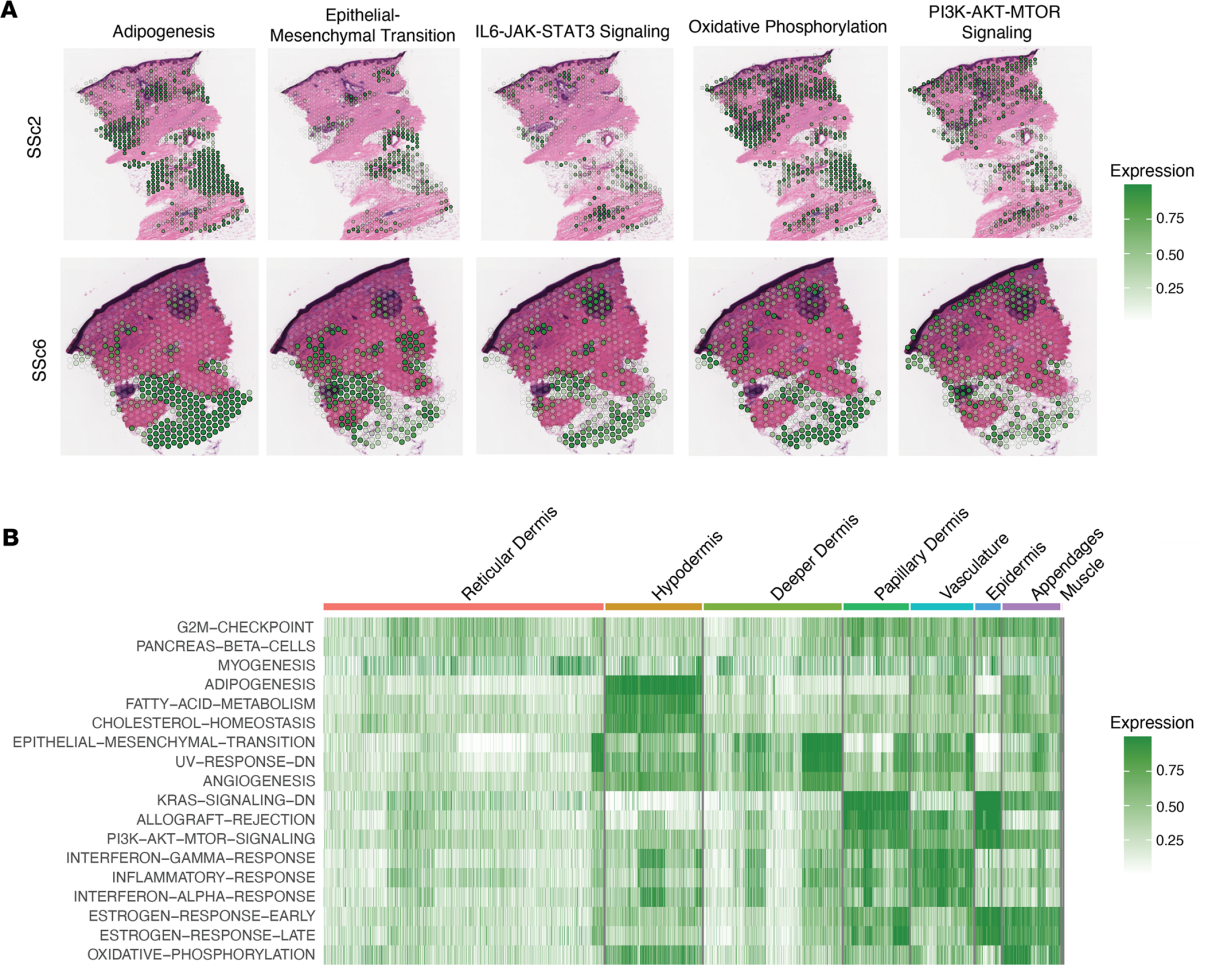
Spatial transcriptomic analyses reveal enrichment of PI3K pathway in distinct anatomic niches. (**A**) Hallmark gene set enrichment in fibroblasts only using VAM; darker spots represent greater enrichment. (**B**) Most enriched hallmark pathways across the anatomical regions of skin (ranked by logFC from Wilcoxon’s rank-sum test).

To investigate population differences in key cell types in the skin, we performed subclustering analyses to assess transcriptomic shift differences between HC and SSc samples. Upon fibroblast and immune cell subclustering, we observed major transcriptomic shifts between SSc and HC samples based on differential expression and chromatin accessibility in the fibroblast and myeloid cell subpopulations (Figures 2 and 3).

We did not observe statistically significant changes in SSc samples of keratinocytes or endothelial cells. No major population shifts or proportional difference in canonical keratinocyte subpopulations (spinous, basal, supraspinous, and epithelial) on a per-sample basis were evident (Supplemental Figure 1, D–G). Subclustering of endothelial cells identified specific subclusters with high expression of fibrotic markers, *ACTA2*, *COL1A1*, and *TAGLN* (Supplemental Figure 1, H–J) but did not exhibit a clear progression through RNA pseudo-time (Supplemental Figure 1K). These data support previous findings and suggest there may be 2 lineages: self-renewing endothelial cells and endothelial cells that become fibrotic (Supplemental Figure 1, K–N) (4).

To map gene expression to specific tissue regions, we performed spatial transcriptomic sequencing on all SSc sections using the 10X Genomics’ Visium platform. After quality-control steps, we found 7,246 spots, with an average of 2,757 unique genes and 9,781 total transcripts per spot. We deconvoluted each spot by the major cell types detected in the snMultiome data. The estimated proportion of each cell type for each spot was displayed on the tissue (Figure 1G), and all cell types were combined as a pie plot for each tissue section (Supplemental Figure 2). Keratinocytes were enriched in the dermis and hair follicles. Fibroblasts were found primarily in the dermis and hypodermis. Immune cells, including myeloid cells, T cells, and B cells, were found to be aggregated around hair follicles, epidermal appendages, and the vasculature. These broad trends were observed in all 10 spatial transcriptomic sequencing sections, with some variation in abundance and enrichment on a per-patient basis (Supplemental Figure 2). Some of the more overtly fibrotic samples, such as SSc9, had challenges in this deconvolution step, and many primary fibroblast-enriched areas were identified as smooth muscle cells due to their more fibrotic components (Supplemental Figure 2I). This classification was later improved through hierarchical deconvolution of the fibroblast-only spots (Figure 3, A and B).

### Growth factors and downstream signaling mediators drive cell survival and differentiation in SSc fibroblasts.

Given the unique nature of this single-nuclei early-disease dataset, we characterized the transcriptomic changes in SSc fibroblasts compared with HC fibroblasts. This shift is evident in the fibroblast subclustering (Figure 2, A–C). Subclustering recaptured key fibroblast subpopulations previously identified by canonical markers, such as *SFRP2^hi^*, *CCL19^hi^*, *COCH^hi^*, and *COL8A1^hi^* (Figure 2, D and E, and Supplemental Figure 3A). In this dataset, we did not observe a significant proportional difference in *SFRP2^hi^* fibroblasts between SSc and HC samples, contrary to other reports (Figure 2C and Supplemental Table 4). However, SSc nuclei were enriched in clusters with high expression of *C3*, *COL8A1*, and *APOD*, or in fibroblasts 0, 7, and 9, respectively (2-tailed *t* test, adjusted *P* values 0.001, 0.009, and 0.04, Supplemental Table 4). Fibroblast 7 expressed high levels of myofibroblast genes *ACTA2*, *SFRP4*, *COL8A1*, *POSTN*, and *COMP* (Figure 2, D and E, Supplemental Figure 3A, and Supplemental Table 5). Consistent with previous studies, this myofibroblast population was closely associated, both graphically and through gene expression profiles, with the adjacent *SFRP2^hi^* fibroblast 6 (4). The *SFRP2^hi^* and *SFRP4^hi^* fibroblast clusters showed enrichment of profibrotic pathways, such as ECM and TGF-β signatures (Supplemental Table 6), aligning with prior literature (2).

Among the fibroblast subclusters that were composed mainly of SSc-derived fibroblasts, fibroblast 0 (*C3^hi^APOE^hi^*) was more likely to be enriched with inflammatory gene signatures, such as the Hallmark IFN-α response, IL-6/JAK/STAT, and complement pathways, which we believe is a potentially novel finding (Figure 2F and Supplemental Table 6) (2–4). Similar clusters were identified in other studies, referred to as perivascular fibroblasts, which have been implicated in fibrotic disorders as immunomodulatory (21, 22).

Pathway analysis showed a genome-wide transcriptomic shift and activation of SSc fibroblasts compared with HC fibroblasts. This shift demonstrated an increased TGF-β signaling and epithelial-mesenchymal transition, as shown by the enrichment of key hallmark pathways (Figure 2F). SSc nuclei also exhibited considerable enrichment of PI3K/AKT/mTOR signaling, oxidative phosphorylation, and the IL-6/JAK/STAT3 signaling pathways (Wilcoxon’s rank-sum test, adjusted *P* value 1.17 × 10^–253^, 1.41 × 10^–95^, and 1.06 × 10^–151^) (Figure 2F and Supplemental Table 7). Further differential expression of genes within the Hallmark PI3K/AKT pathway revealed a distinct signature of pathway genes that was increased in SSc across all fibroblast subsets (Supplemental Figure 3C and Supplemental Table 8). Gene set variation analysis (GSVA) using the Reactome database revealed that many gene sets uniquely expressed in the SSc fibroblasts are predominantly involved in growth factor response and signaling (Figure 2G and Supplemental Table 9).

When performing Gene Ontology (GO) term analysis with Kyoto Encyclopedia of Genes and Genomes (KEGG) gene sets, we identified significant PI3K enrichment as well as ECM receptor signaling in all SSc fibroblast populations (Supplemental Figure 3B and Supplemental Table 10). There was also significant enrichment of collagens and growth factor receptors among the genes contributing to the KEGG and Hallmark gene set enrichment. Pathway analysis indicated that a commensurate signal cascade and metabolic pathways were enriched in SSc samples within this cohort (Supplemental Figure 3, B–D). Transcriptomic alterations indicated a global shift within the fibroblast subclusters, suggesting that targeting pathways such as the PI3K/AKT/mTOR and IL-6/JAK/STAT pathways may reduce disease progression in skin fibrosis.

The snATAC-Seq data can be used to infer transcription factor binding, which can, in turn, support pathway activity driving SSc skin fibrosis. SSc fibroblasts had a distinct profile of transcription factor binding largely dominated by ETS family members, consistent with previously reported findings that show the ETS family is highly active in SSc endothelial cells (Figure 2H and Supplemental Table 11) (9). TGF-β–induced transcription factor activity, SMAD2/SMAD3, was also observed in SSc samples and is also regulated by induction of the PI3K/AKT/mTOR pathway (Supplemental Figure 3D, Supplemental Table 11). Transcription factors downstream of the Hippo and PI3K pathways, such as RUNX1, CREB, and EGR1 binding, were enriched in SSc fibroblasts (Supplemental Table 11) (4). Broken up by cluster, we observed that our SSc-dominant fibroblast 7, *COL8A^hi^*, has a unique transcription factor activity signature dominated by FOS-related and JUN-related transcription factors (Figure 2I and Supplemental Table 12). Enrichment of these transcription factors indicates a differentiating population consistent with our understanding of myofibroblasts or cells undergoing a transition toward a myofibroblast phenotype (23).

Extended analyses of fibroblast populations highlight alterations within this dataset. A comparative analysis of ARA^+^ patient samples showed minor changes in fibroblast compartments based on autoantibody status. Notably, there were few significant subcluster differences among HC, ARA^–^, and ARA^+^ samples (Supplemental Figure 3E). Differential expression analysis based on ARA status in SSc fibroblasts revealed only a small number of differentially expressed genes (Supplemental Figure 3F and Supplemental Table 13). Among these, ARA^–^ samples were enriched in *APOE* and *C3*, genes associated with more immunomodulatory fibroblasts. Additionally, further characterization of fibroblast subtypes enriched in HC samples indicated no substantial or functional differences in these cells (Supplemental Figure 3, G and H).

RNA-trajectory analysis of the fibroblast population showed multiple potential lineages contributing to this collagen-producing niche. Trajectory analysis showed several branches across pseudo-time within these clusters (Supplemental Figure 4, A and B). Designating a central population as the root node resulted in a pseudo-time endpoint associated with increased expression of myofibroblast markers, such as *SFRP4* and *PRSS23*, across all trajectory branches (Supplemental Figure 4C). When comparing divergent branches, SFRP2*^hi^* fibroblasts exhibited increased *PRESS23* expression along their branch (Supplemental Figure 4, D and E). The fibrotic branch (branch 1) showed decreased *CXCL12* and *APOE* expression throughout its pseudo-time. Conversely, the other primary branch (branch 2) began with high *CXCL12* and *APOE* levels, which remained elevated throughout pseudo-time (Supplemental Figure 4F). These analyses support previous findings that *SFRP2^hi^* fibroblasts serve as progenitors to *SFRP4^hi^* myofibroblasts. Additionally, these analyses suggest that divergent fibroblast lineages exist in fibrosis and may play various roles in disease pathology.

Finally, we examined the enrichment of key Hallmark pathways in fibroblasts across the spatial context of the matching tissue sections in Visium spots that contained only fibroblasts (Figure 3). To achieve this, we first performed a hierarchical deconvolution filtering the Visium spots based on the estimated presence of 2.5% or more of fibroblasts. We then probed only those spots for pathway enrichment. As expected, we found that the hypodermis was enriched in the adipogenesis pathway (Figure 3, A and B). When probing the spatial sections for pathway-based enrichment, we found that the PI3K/AKT/mTOR signaling pathway was specifically enriched in the papillary dermis, vasculature, and epithelial appendages (Figure 3, A and B, and Supplemental Table 14). The correlation of cell types localized within the same region indicated potential interactions and supported pathway activation in each of these areas (Supplemental Figure 5A). The enrichment of key pathways aligned with the localization of fibroblasts 0 and 3 near the vasculature regions in spatial data of the dermis (Supplemental Figure 5, B and C). This growth factor response in the papillary dermis in early-stage disease is noteworthy, considering the documented structural disruption of the papillary dermis during the progression of SSc skin fibrosis (24).

### SSc-specific macrophage activation in early fibrotic skin.

Patients with SSc with active disease are often treated with immunosuppressants, such as mycophenolate mofetil and methotrexate (6), which can significantly alter immune cell composition and activity. By studying samples from treatment-naive patients, our analysis captures the endemic immune landscape in the early stages of disease. This enables a clearer understanding of the pathogenic mechanisms in SSc skin fibrosis and provides insight into early disease pathogenesis. Previous in vitro studies have demonstrated a critical role for macrophages in activation of SSc fibroblasts, while experimental studies in different model systems and meta-analyses have shown a connection between profibrotic macrophage signatures and SSc skin fibrosis (25–29).

**Figure 4:**
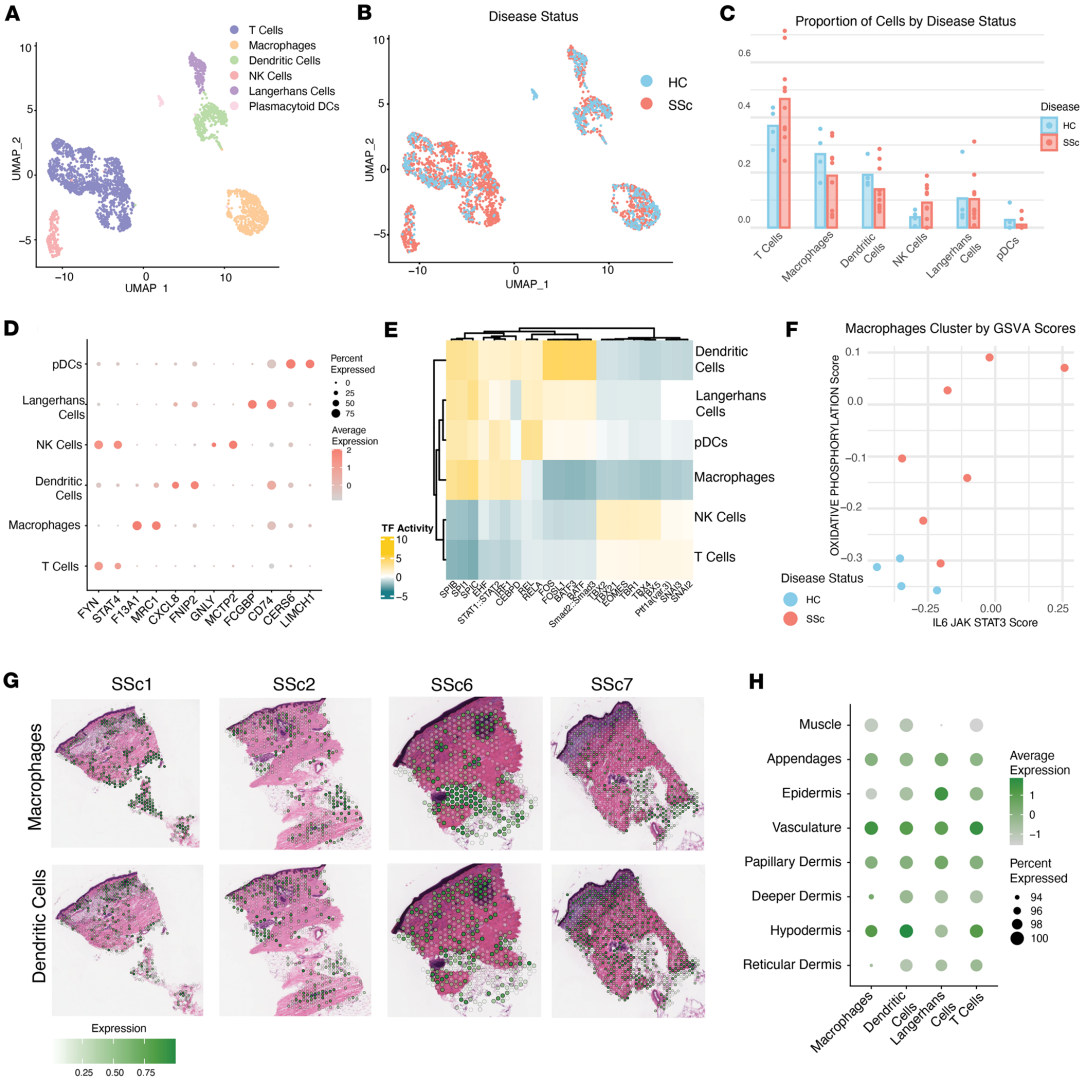
Immune compartment shows macrophage activation in SSc samples. (**A**) Multimodal UMAP shows T cell and myeloid cell subpopulations. (**B**) Multimodal UMAP shows the broad SSc and HC nuclei distribution in the subclustered immune cells. (**C**) The proportion of each immune cluster per patient in SSc and HC. There are no significant differences in the proportion of immune cells (2-tailed *t* test). (**D**) Two of the most differentially expressed RNA transcripts from each immune cell type (Wilcoxon’s rank-sum test). (**E**) The most active transcription factors per immune subtype. Key canonical transcription factors such as SPIC, STAT1/STAT2, SPIB, and CEBPD were significantly active in macrophage populations (Wilcoxon’s rank-sum test, adjusted *P* values ≤ 0.05). (**F**) A scatter plot for each sample of the enrichment scores for the pseudo-bulked macrophages shows significant differential variation in hallmark gene sets, oxidative phosphorylation, and IL-6/JAK/STAT3 signaling in the SSc samples (Wilcoxon’s rank-sum test compared by disease status, all *P* values ≤ 0.05). (**G**) Immune cell gene set enrichment in the immune cells only contains spots across the tissue sections, using VAM. (**H**) Most enriched immune cell gene sets across the anatomical regions (Wilcoxon’s rank-sum test).

When parsing the immune compartments of these samples, we found no significant differences in the proportion of SSc T cells or myeloid cells to HC cells per sample; however, differential expression analyses revealed differences in SSc immune cell activity (Figure 4, A–C, and Supplemental Table 15). We observed that nearly all of the B cells originated from a single sample; this sampling difference has been observed in other studies where the majority of B cells were derived from single-patient samples (Figure 1D and Supplemental Table 3) (4). Subclustering the immune cell compartment, we identified key immune cells: T cells, NK cells, DCs, plasmacytoid DCs (pDCs), Langerhans cells, and macrophages, with their significantly enriched genes matching canonical markers (Figure 4D and Supplemental Table 16). We confirmed cell type and activity using snATAC-Seq data by examining the differentially accessible peaks near canonical genes of interest, such as *MRC1*, *CD1C*, *GZMB*, and *IL32* (Supplemental Figure 6A and Supplemental Table 17). Using accessible ATAC peaks, we inferred differences in transcription factor activity between cell types (Figure 4E and Supplemental Table 18). The transcription factor activity in these samples aligned with the expected transcription factor binding in restricted cell types. Specifically, we observed high SPIC activity in macrophages and DCs and high TBX family activity in T cells (Figure 4E).

When comparing transcriptomic and epigenetic differences among dermal samples, we observed a genome-wide shift in activation within the SSc macrophage compartment. Some transcription factors that had increased activity in SSc macrophages were CEBP-related (CEBPD, CEBPA, CEPBG) that were more strongly associated with activated macrophages (Figure 4E, Supplemental Figure 6B, and Supplemental Table 17). Furthermore, we could distinguish SSc macrophages from their HC counterparts by the enrichment of IL-6/JAK/STAT3 and oxidative phosphorylation signaling (Figure 4F and Supplemental Table 19) (25). This showed a transcriptomic shift specific to SSc macrophages, which may contribute to their inflammatory and profibrotic activation states. Ligand-receptor analyses found differences that may contribute to disease, namely, increased macrophage inhibitory factor (MIF) and amyloid precursor protein (APP) interactions from macrophages to T cells, which may indicate increased inflammatory responses (Supplemental Figure 6, C and D). When examining the expressed growth factors and signaling mediators within each population, we observed that the macrophage clusters express profibrotic factors, including *TGFB*, *PDGF*, *FGF*, and *IL6*.

Finally, using the spatial transcriptomic data, we observed enrichment of immune cell types across the dermis. Macrophages were significantly enriched in the hypodermis, epidermal appendages, papillary dermis, and vasculature (Figure 4, G and H, and Supplemental Table 20). This spatial enrichment of profibrotic macrophages coincides with the enrichment of the fibroblast pathway in the PI3K/AKT/mTOR pathway in these regions (Figure 3, A and B).

### Spatial, transcriptomic, and functional evidence for TGF-β– and PDGF-mediated fibroblast activation in the SSc vasculature niche.

**Figure 5:**
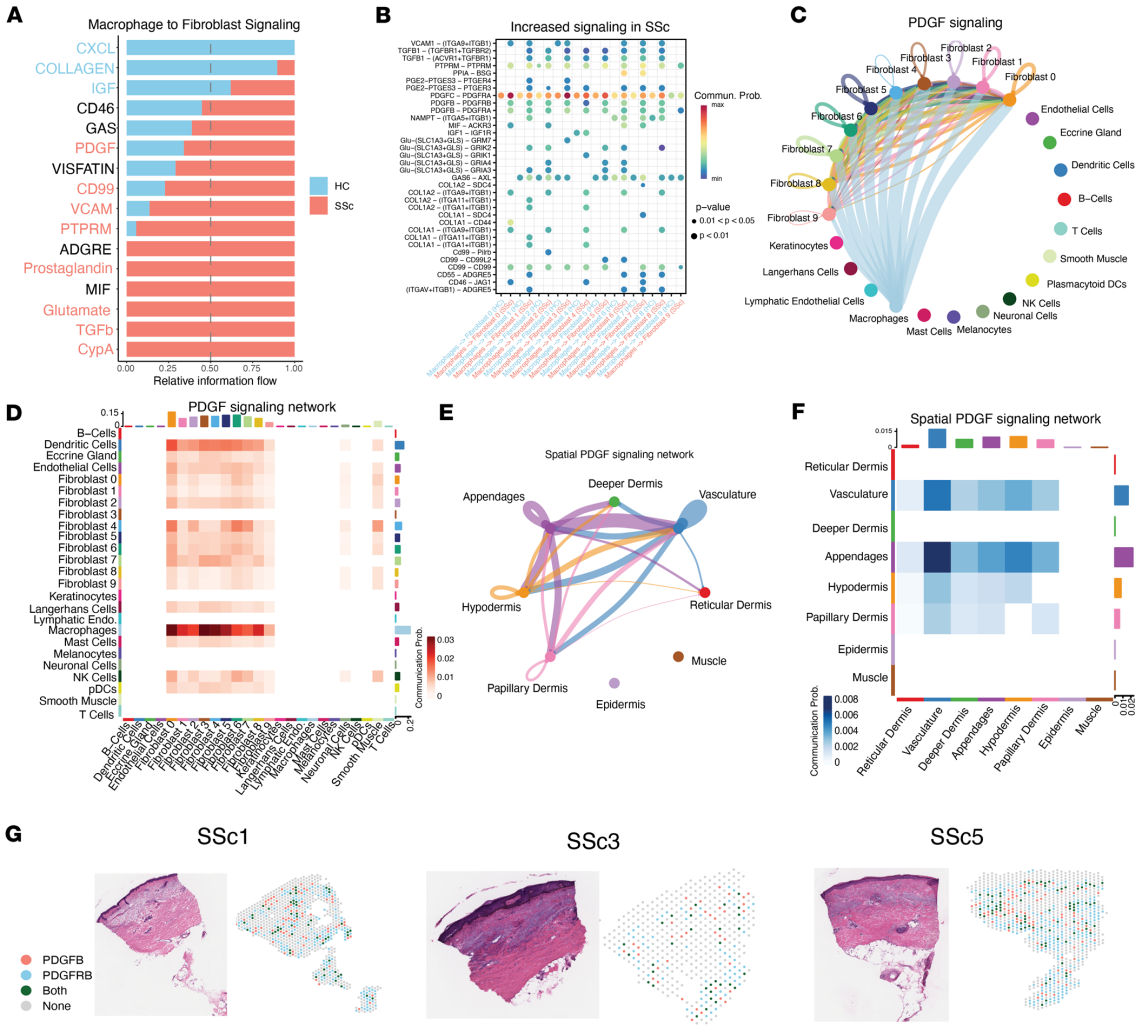
Ligand-receptor analysis demonstrates the enrichment of PDGF signaling from macrophages to fibroblasts. (**A**) The relative information flow plot shows a shift in the signaling of macrophages to fibroblasts by the expression of macrophage ligands and their respective receptors on fibroblasts. Significant differences in the interactions are colored by coloring the pair by the driver condition (paired Wilcoxon’s test). (**B**) Dot plot showing the communication probability between individual receptor-ligand pairs between macrophages and each fibroblast subcluster comparing HC and SSc interaction. Alternating interactions show increased probability and expression of individual receptor-ligand pairs in TGF-β and PDGF signaling pairs (Wilcoxon’s rank-sum test). (**C**) A chord diagram shows the direct signaling of all PDGF ligands from all myeloid cells to all fibroblast subclusters, with a more substantial signaling probability of macrophages than dendritic signaling to fibroblast subclusters. (**D**) Heatmap shows PDGF ligand-receptor interactions in all cells, including the immune and fibroblast subclusters. The highest probability of PDGF signaling interactions is observed between macrophages and all fibroblast subclusters. (**E**) A chord diagram shows the signaling of all PDGF ligands within the major anatomical structures of the skin. The most probable interactions are the epidermal appendages and vasculature signaling to the layers of the dermis. (**F**) The heatmap shows PDGF ligand-receptor interactions in the major structural regions. The highest probability of PDGF signaling interactions is observed between epidermal appendages and the dermal layers. Probable interactions are also observed in the papillary dermis and the hypodermis. (**G**) Representative histology Visium spot sections showing expression of PDGFB and PDGFRB with coexpression in spots.

PDGF emerged as the most probable and statistically significant interaction between macrophages and fibroblasts through ligand receptor analyses (Figure 5A, Supplemental Figure 7A, and Supplemental Tables 21 and 22). When comparing the contributions of macrophage signaling to each fibroblast subcluster, we observed that secretion of PDGFC and PDGFB by macrophages was predicted to interact with PDGFRA and PDGFRB in fibroblasts across both SSc and HC tissues. This interaction was significantly more probable in cells derived from SSc skin biopsies, with PDGFB ligand signaling to PDGF receptors observed exclusively in SSc cells (Figure 5B and Supplemental Figure 7, B and C). This result suggests macrophage-driven fibroblast activation in SSc skin. In contrast, the dominance of CXCL and collagen-based signaling in HC samples is consistent with the role of macrophages in maintaining and regulating skin homeostasis and tissue remodeling (30).

Although PDGF was among the most probable significant interactions, we also observed a wide range of previously identified profibrotic pathways. For example, fibrosis-dependent TGF-β signaling between and within fibroblasts and immune cells has been well-documented (15, 25). We consistently observed TGF-β crosstalk between SSc macrophages and DCs with SSc fibroblasts (Supplemental Figure 7D and Supplemental Table 21). However, prominent signaling interactions between SSc myeloid cells and fibroblasts were not limited to TGF-β signaling in SSc samples, as pathways associated with prostaglandin, glutamate, CD99, and VCAM signaling were also identified (Figure 5, A and B, Supplemental Figure 7, E–I, and Supplemental Table 22). VCAM has been previously implicated in SSc pathogenesis, with elevated serum levels reported in patients with SSc relative to controls (31). Although these do not necessarily correlate with prognosis, dermal VCAM signaling may contribute to downstream PI3K/AKT activity (32). These pathways, while significant in our interaction analysis, had relatively low expression in macrophages and fibroblast subtypes, which makes their probability of interaction low in this dataset.

Next, we confirmed the macrophage-specific signaling of PDGF to fibroblasts. A chord diagram demonstrates the increased probability of sending PDGF by macrophages to each of the fibroblast subclusters (Figure 5C). The nuance of this interaction was revealed by the quantitative differences in the probability of the PDGF signaling network (Figure 5D). The network heatmap showed an increased probability of signaling between macrophages and fibroblasts, with the highest probability of interactions occurring between macrophages and fibroblast clusters 0 and 3. Notably, the gene expression of fibroblast clusters 0 and 3 is more immunomodulatory (*C3^hi^* and *CCL19^hi^*). This interaction underlines the significant proinflammatory and profibrotic role of macrophage signaling in SSc skin disease. Our analyses support a model in which signals transduced by macrophages induce activation of inflammatory fibroblasts, which then recruit more immune cells and activate other fibroblast subtypes to produce additional fibrotic factors.

PDGF signaling interactions are recapitulated within the Visium spatial transcriptomic data to provide an anatomic context. Prediction of PDGF network interactions revealed that PDGF ligands are primarily derived from the vasculature, epidermal appendages, hypodermis, and papillary dermis, matching the enriched macrophage signatures within these regions (Figure 4H and Figure 5, E and F). The correlations between fibroblasts, endothelial cells, and myeloid cells highlight the likelihood of finding them together in a specific niche as observed in the spatial transcriptomic data (Supplemental Figure 5A). When comparing across SSc tissues, we observed restriction of individual expression of PDGF receptors or ligands to the epidermal skin appendage and vasculature regions, and notable coexpression within these key regions (Figure 5G). These spatial transcriptomic data highlight the importance of macrophage activation in the initiation of early profibrotic PDGF signaling.

**Figure 6:**
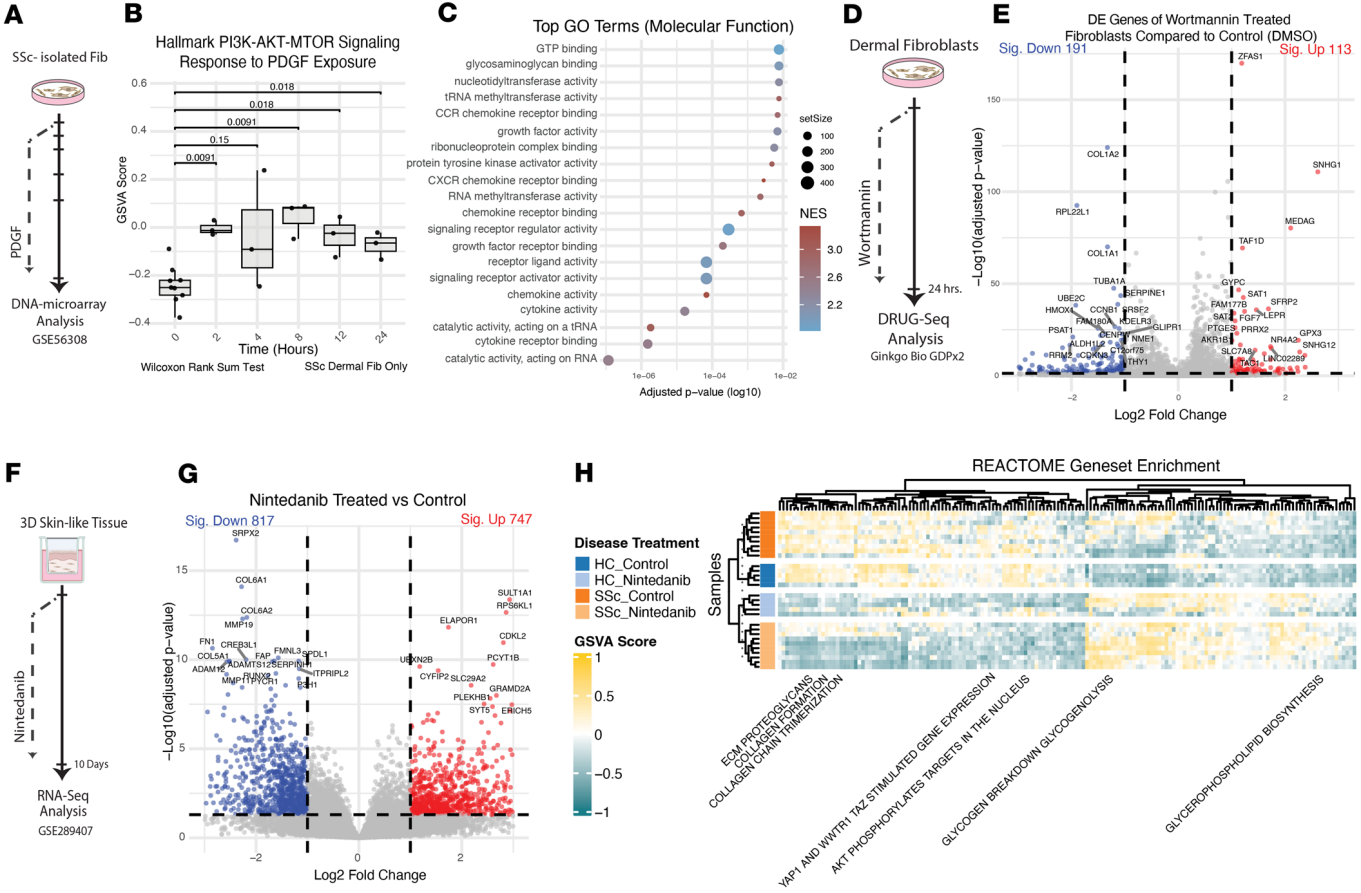
In vitro studies highlight the fibroblast PDGF response and demonstrate the efficacy of PDGFR and PI3K inhibitors. (**A**) Experimental design of PDGF exposure to the SSc-isolated dermal fibroblasts. (**B**) Box-and-whisker plots of enrichment scores for Hallmark PI3K/AKT/mTOR pathway. Comparison to baseline shows a significant increase in GSVA score for most time points, with the largest differences after 2 and 8 hours of PDGF exposure (Wilcoxon’s rank-sum test, *P* value = 0.0091). The center line of the box plot indicates the median value; the box spans the first and third quartiles; whiskers show the range, excluding outliers. (**C**) GO term analysis of gene sets shows the molecular functions overrepresented in fibroblast lines 8 hours after PDGF treatment versus the baseline. Sorted by adjusted *P* value (GSEA, permutation test). (**D**) Experimental design of dermal fibroblasts treated with wortmannin. (**E**) Volcano plot with differentially expressed genes comparing 3,000 nM wortmannin treatment with DMSO-treated dermal fibroblasts. The most downregulated and upregulated genes by LogFC and adjusted *P* value are listed (Wald’s test). (**F**) Experimental design of 3D skin-like tissues treated with nintedanib from GEO GSE289407. (**G**) Volcano plot with differentially expressed genes comparing all nintedanib-treated tissues with control (vehicle-treated). The top most downregulated and upregulated genes by LogFC and adjusted *P* value are listed (Wald’s test). (**H**) Reactome gene sets enriched compared by sample of origin and 3D skin-like tissue treatment. Only gene sets with significant differences by ANOVA are displayed.

To validate the downstream effects of PDGF ligands and PDGF inhibition on fibroblasts, we analyzed previously generated bulk RNA datasets. We examined a DNA microarray dataset generated from independent SSc fibroblast cell lines treated with 30 ng/mL PDGF-BB (NCBI’s Gene Expression Omnibus [GEO] GSE56308, Figure 6A) (33). GSVA revealed a significant increase in the Hallmark PI3K pathway following longitudinal PDGF exposure, peaking at approximately 8 hours and remaining elevated for 24 hours (Figure 6B and Supplemental Table 23). Differential gene expression analysis comparing SSc fibroblasts collected after 8 hours of PDGF treatment compared with untreated levels revealed significantly elevated mRNA levels of key PI3K pathway genes, including *MYD88*, *UBE2N*, *PDK1*, *TIAM1*, *PITX2*, and *SLC2A1* (Supplemental Figure 8A and Supplemental Table 24). GO term analysis of the differentially expressed pathways using GO-Molecular Function showed enrichment of “cytokine activity,” “signal receptor binding/activity,” “growth factor activity,” and “kinase receptor activity,” thus demonstrating PDGF-driven fibroblast activation that aligns with early untreated fibroblasts in patient skin (Figure 6C and Supplemental Table 25).

After confirmation of a PDGF-driven response in SSc fibroblasts, we interrogated drug treatments to mitigate the effects of PDGF. Using a bulk RNA-Seq dataset analyzing cellular responses to a panel of common compounds, we were able to compare the gene expression profiles of dermal fibroblasts in response to increasing concentrations of wortmannin, a PI3K inhibitor (Ginkgo Bio dataset GDPx2, Figure 6D) (34). This inhibitor reduced the enrichment of the PI3K pathway across all concentrations (Supplemental Figure 8B). We observed that *COL1A1*, *COL1A2*, and other known profibrotic genes were significantly downregulated by wortmannin treatment (Figure 6E and Supplemental Table 26), indicating that PI3K inhibitors can have broad downstream effects on fibroblasts in fibrotic conditions.

We analyzed experiments that treated 3D skin-like tissues with nintedanib and assessed the impact on PI3K pathway activation and ECM production. We reprocessed publicly available data (GEO GSE289407) of 3D skin-like tissues derived from control or SSc dermal fibroblasts treated with nintedanib or vehicle control (Figure 6F). Differential expression analysis of nintedanib-treated tissues compared with vehicle control demonstrated a marked decrease in ECM markers such as *COL6A2*, *FN1*, and *SERPINEH1* (Figure 6G and Supplemental Table 27). GSVA showed changes in ECM proteoglycan, collagen formation, and collagen chain trimerization between HC- and SSc-derived tissues; there was a notable reduction in these pathways with nintedanib treatment in both HC and SSc samples (Figure 6H, Supplemental Figure 8, C and D, and Supplemental Table 28). AKT phosphorylation targets, which are directly downstream of PI3K pathway activation, were lost with nintedanib treatment. Together, these data demonstrate that targeting the PDGF and PI3K pathways can reduce key markers of fibrosis.

### Spatial neighborhoods show myeloid cells and proinflammatory fibroblasts create a distinct vasculature niche in early SSc skin.

**Figure 7:**
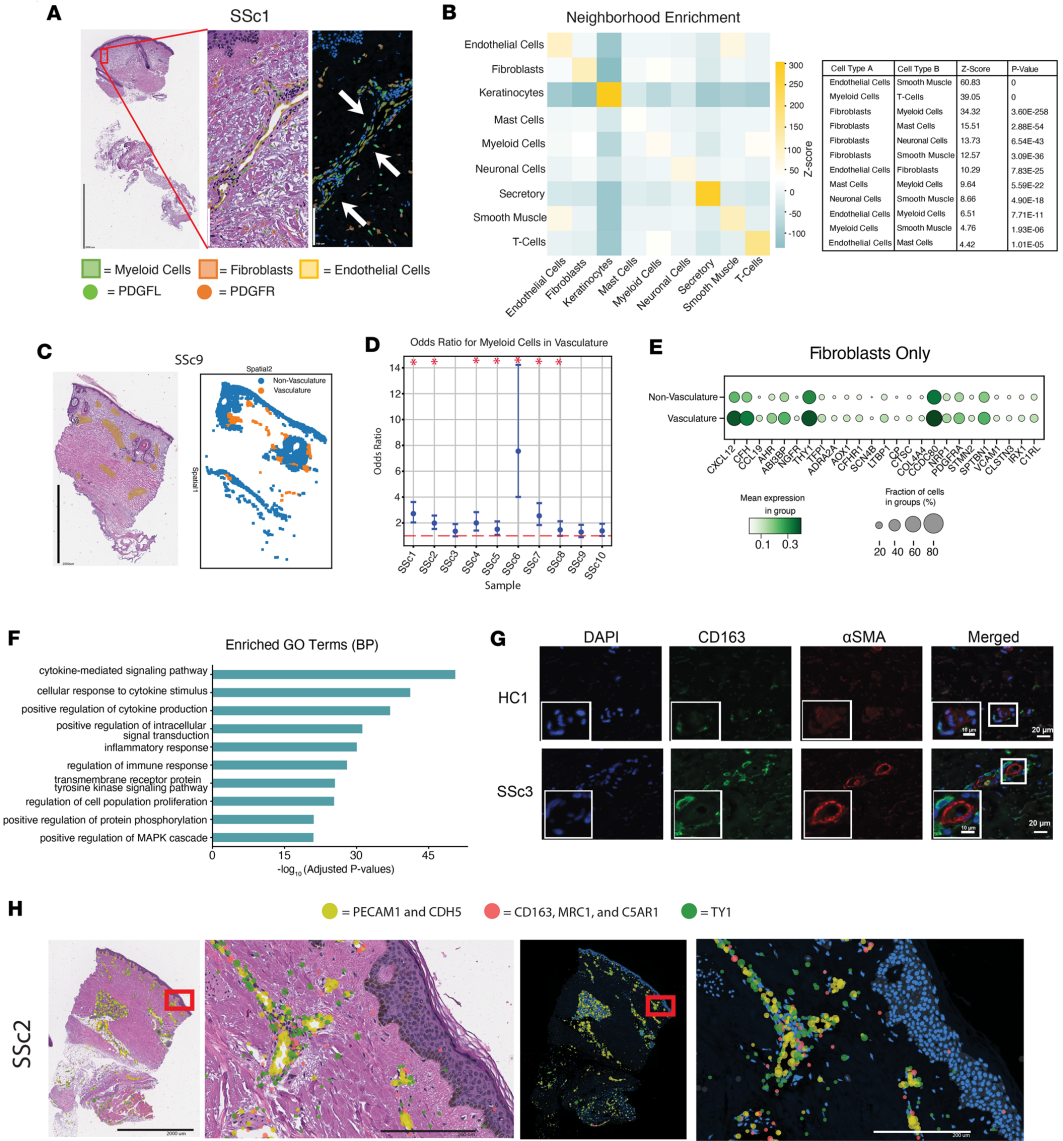
Single-cell spatial analysis demonstrates distinct, cytokine-rich neighborhoods near vasculature in SSc skin. (**A**) Representative Xenium images of fibroblasts (orange), myeloid cells (green), and endothelial cells (yellow). Fibroblasts express PDGF receptors; myeloid cells were more likely to have PDGF ligand expression. PDGF receptors (PDGFR; orange dots), all PDGF ligands (PDGFL; green dots); scale bars: 1,000 μm, 200 μm, and 200 μm (left to right); arrows: infiltrating myeloid cells near fibroblasts. (**B**) Neighborhood enrichment analysis quantifies likelihood of spatial proximity between cell types;10 most common cell neighbors, disregarding self, shown in table; *z* scores indicate strength of overrepresented neighbor (*z* score and *P* values from *z* test from randomized cell spatial distribution). (**C**) Representative images with manually annotated vasculature regions overlaid on histology sections and cellular density with region identification. (**D**) Odds ratio calculations across all sections show in most sections, myeloid cells more likely to be found in annotated vasculature regions. (**E**) Differential expression analysis shows top 25 genes (sorted by LogFC, Wilcoxon’s rank-sum test, adjusted *P* values < 0.05) overexpressed in vasculature regions compared with other sections. Expected genes (e.g., *EPAS1* and *PECAM1*) demonstrate specificity of endothelial cells in these regions. Other genes of interest (e.g., *CXCL12*, *TGFBR2*, and *CCL14*) indicate distinct proinflammatory environment. (**F**) GO term analysis indicates pathway-level enrichment of key terms: cytokine-mediated signaling, cellular response to cytokine stimulus, and regulation of immune response (sorted by adjusted *P* value, GSEA, permutation test). (**G**) Immunofluorescence showing neighboring of CD163-positive cells and α-SMA–positive cells in SSc skin compared with HC tissues. Images represent *n* = 2 for HC and *n* = 3 for SSc. Scale bar: 20 μm. (**H**) Xenium overlay of selected sections. *PECAM* and *CDH5* pseudo-colored yellow to identify endothelial cells; *CD163*, *MRC1*, and *C5AR1* are combined and pseudo-colored red to identify macrophages; and *THY1* pseudo-colored green to identify fibroblasts. H&E overview and inlay displayed alongside DAPI (blue) counterstain overlay and inlay of same regions. Scale bars: 2,000 μm (overview) and 200 μm (inlay).

Our findings led us to interrogate these cell-cell interactions in a high-resolution, single-cell space. To visualize and measure these interactions, we performed high-resolution in situ analysis combined with cellular morphology stains on all 10 SSc samples using the 10X Genomics’ Xenium platform. We tested the hypothesis that fibroblasts and myeloid cells, specifically macrophages and DCs, interact in specific regions of the dermis, providing insight into cooperative mechanisms of pathogenesis. Gene expression was determined for a panel of 5,000 genes in 64,844 cells across FFPE sections of SSc skin samples on which snMultiome and 10X Genomics’ Visium analyses were performed. This analysis identified 9 cell types. Cell type information and gene expression were overlaid on the H&E-stained tissue sections. In addition, immunofluorescent staining for nuclei (DAPI), cell boundaries (ATP1A1/CD45/E-cadherin mix), intracellular RNA (18S), and interior proteins (α-SMA/vimentin mix) were performed for structural visualization and cell segmentation. We specifically examined the expression of PDGF ligands (PDGFL, including PDGFA, PDGFB, and PDGFC) and PDGF receptors (PDGFR, including PDGFRA and PDGFRB), as shown in a representative sample (Figure 7A). The higher magnification region of the image highlights a region of vasculature. Cell types were identified by cell clustering, canonical gene expression, and automated cell type annotation from prior multiome data. Myeloid cells are outlined in green, fibroblasts are outlined in red, and endothelial cells are outlined in yellow. On this high-resolution platform, we observed increased numbers of myeloid cells expressing high amounts of PDGFL (green dots) near the vasculature, identified by endothelial cells. We also observed fibroblasts expressing high amounts of PDGF receptors (red dots) in the same region, indicating colocalization of these cells around the vasculature, one of the earliest sites of injury in SSc.

To quantify myeloid cell and fibroblast colocalization near the vasculature in SSc skin, we performed a neighborhood enrichment analysis to identify the nearest neighbors for each cell type within the entire tissue section. In accordance with expectations, the nearest neighbor for each cell type was itself (Figure 7B and Supplemental Table 29); that is, keratinocytes were most likely to be near other keratinocytes. We therefore excluded these self-driven neighborhoods from further analyses and identified 16 significant overrepresented cell-cell neighbors. The most observed cell neighborhoods aligned with biological expectations; for example, the nearest neighbor of endothelial cells was smooth muscle cells (Figure 7B). Notably, the third most common neighboring pair was fibroblasts and myeloid cells (*z* score 34.3, *P* value = 3.60 × 10^–258^). Among this list, endothelial cells were also found near both fibroblasts and myeloid cells. This neighborhood enrichment highlights a distinct anatomic niche within the skin, composed of endothelial cells, fibroblasts, and myeloid cells.

To confirm the finding that myeloid cells are enriched near the vasculature, each tissue section image was segmented to isolate vasculature regions (Figure 7C and Supplemental Figure 9). An odds ratio test was performed to identify the likelihood of finding myeloid cells within the vasculature regions compared with the rest of the tissue section, and 70% of samples showed a 1.5 times increase in myeloid cells in the vascular regions compared with the nonvascular regions of the tissue (Figure 7D and Supplemental Table 30). The specificity of the vasculature segmentation was confirmed by calculating the odds ratio for endothelial cells. This analysis found that endothelial cells were more than 2.5 times more likely to be found in the selected vascular regions of the dermis, and fibroblasts were also about 1.5 times more likely to be found in these sections (Supplemental Figure 10, A and B, and Supplemental Tables 31 and 32). These data support the finding that myeloid cells and fibroblasts occupy a localized cellular niche near the vasculature in SSc skin.

Differential expression analysis was performed to characterize the transcriptomic changes in vascular-associated myeloid-fibroblast neighborhoods. Gene expression was compared between cells within the prescribed vasculature regions relative to the cells outside of it. Differentially expressed genes, such as *EPAS*, *PECAM1*, and *ENG*, indicated endothelial cell enrichment (Supplemental Figure 10C and Supplemental Table 32). Additional differentially expressed genes suggest enrichment of specific fibroblast subclusters and myeloid cells (macrophages and DCs) within these regions (Supplemental Figure 10, C and D). For example, *CXCL12* and *CCL14* (*P* value < 0.01, Wilcoxon’s rank-sum test), known macrophage chemoattractants, were expressed in key fibroblast subclusters (fibroblasts 0 and 3) in the snMultiome data and were enriched in SSc compared with HC (fibroblasts 0 and 3, Figure 2B). When fibroblasts were analyzed separately by region, many of the same fibroblast markers in the immunomodulatory fibroblasts were found near the vasculature (Figure 7E). This finding is supported by markers indicative of perivascular and fibroblastic reticular cell–like (FRC-like) (F2/F3: perivascular and F3: FRC-like) fibroblasts found near the vasculature in other diseases (21).

GO term analysis was used to identify the pathways overrepresented in the vascular myeloid-fibroblast tissue niche. These regions were characterized by a marked increase in genes associated with cytokine-mediated signaling, cellular response to cytokine stimulus, and the inflammatory response (Figure 7F and Supplemental Table 33). The enrichment for immune-specific pathways further supports the vascular niche as a region of myeloid-fibroblast crosstalk in SSc skin.

Finally, we performed immunofluorescent staining on 3 diffuse cutaneous SSc and 2 HC skin biopsies. Figure 7G shows representative images of an HC and an SSc sample. We used CD163 to identify macrophages and α-SMA to identify activated myofibroblasts and pericytes near the vasculature. Nuclei were identified by DAPI staining. In SSc samples, CD163-positive cells were frequently observed near vascular regions. In addition, α-SMA–positive cells identified pericytes, which was less common in HC samples (Figure 7G and Supplemental Figure 11A). These data from immunofluorescence staining were corroborated by the Xenium data, which found that fibroblasts marked by *THY1*^+^ were found in a niche with profibrotic macrophages marked by *CD163*^+^*MRC1*^+^*C5AR1*^+^ and were localized near the vasculature as indicated by *PECAM1*^+^*CDH5*^+^ (Figure 7H and Supplemental Figure 11B).

Taken together, the spatial analyses coupled with the snMultiome results support the hypothesis that myeloid-fibroblast interactions are localized near the vasculature in SSc skin and form a unique proinflammatory niche that is key to driving the early vascular pathology observed in SSc.

## Discussion

Efforts to accurately assess the early disease dynamics of SSc dermal fibrosis are challenging due to limited patient resources and a minimal number of patients not receiving therapy. This study used a vertically integrated cohort of untreated patients with early-stage, diffuse cutaneous SSc analyzed by multiple modalities, including snMultiome (snRNA-Seq and snATAC-Seq) and 2 spatial technologies: 10X Genomics’ Visium, providing a full transcriptome at low spatial resolution, and 10X Genomics’ Xenium, which analyzes 5,000 genes at single-cell resolution. The analysis of these data from this group of patients with early-stage SSc allows us to investigate the signaling interactions among and between cells and then to place these cells into their anatomic context within the structure of the skin. We sought to characterize the tissue interactome without confounding influences of immunomodulatory therapies, which are common among many of the other SSc cohorts previously studied.

Although prior studies have focused on more profibrotic myofibroblasts, which we also observed in this study, we observed the prominent activity of an immunomodulatory, perivascular fibroblast population that was found to be enriched within a vascular niche of the skin in close proximity to myeloid cells. Immunomodulatory fibroblasts have been emphasized in other pathological conditions and implicated in disease progression and immune cell recruitment (22, 38, 39). This observation suggests an underrecognized, potentially stage-specific enrichment in fibroblast phenotypes. Our data support previous findings of *COL8A1^hi^* myofibroblasts, which are associated with increased fibrosis and are derived from *SFRP2^hi^* fibroblasts (2, 4). The early activity of a more immunomodulatory fibroblast phenotype may reflect the initial inflammatory phase of SSc pathogenesis, which precedes and then transitions into a fibrotic stage (39); this question can only be answered by a longitudinal time-course analysis using similar approaches.

The enrichment of the PI3K/AKT/mTOR pathway in most SSc fibroblast subclusters indicates an underlying commonality across the immunomodulatory and profibrotic subclusters high in *SFRP2*, *SFRP4*, and *COLA8A1*. Other studies have implicated the enrichment of this pathway in early SSc fibroblasts from ARA^+^ patient samples (5). This study builds on these findings by demonstrating that this pathway was broadly enriched in most early SSc compared with HC fibroblasts. Furthermore, the enrichment of the PI3K pathway has been implicated in SSc and other types of fibrosis (20, 40, 41). These findings are further contextualized through a wound study by Yao et al., where early PDGFR activation in fibroblasts supports fibroblast proliferation and survival, allowing subsequent fibroblast-to-myofibroblast transition (42). Altogether, these findings and previous work indicate that targeting the PI3K signaling cascade in fibroblasts is a promising target for ameliorating fibrosis (17).

Our study supports the hypothesis and findings of prior studies that macrophage activation is a key driver of fibrosis in SSc, and the gene expression we observed in patients with early-stage SSc is consistent with a prior report of CD206- and CD163-positive profibrotic macrophages induced by the SSc-specific microenvironment (25). Here, we demonstrated this activation on a per-sample basis, indicating a commonality among patients with early-stage SSc. Furthermore, we found that the SSc macrophages in this cohort have a significantly different interactome with the fibroblast subclusters. Specifically, the high probability of interaction between TGF-β and PDGF ligands produced by the macrophages is consistent with our prior reports (25), and we found their respective receptors (e.g., PDGFR) on the more immunomodulatory, or “perivascular,” fibroblasts near the vasculature. This pattern of activation is specific to SSc, indicating that the invasion and polarization of these macrophages may drive the survival and activation of immunomodulatory and profibrotic fibroblasts. The findings from this study strongly support the enrichment of a profibrotic and SSc-specific phenotype in the macrophage population that is infiltrating fibrotic skin.

We previously treated fibroblasts with a wide range of profibrotic factors, including TGF-β and PDGF (33, 43). Analysis of the time course of PDGF treatment of HC and SSc dermal fibroblasts confirmed the downstream effects of PDGF ligands, mainly the induction of profibrotic gene expression programs in addition to immunomodulatory programs, on SSc fibroblasts (33). Prior studies in SSc also provide strong evidence that PDGF ligands are overexpressed in SSc skin and in the plasma in multiple patient cohorts (44, 45). We probed the downstream effect of nintedanib treatment on 3D skin-like tissues and found a marked decrease in PI3K pathway activity targets, as well as a significant reduction in ECM production. We also demonstrated that treating dermal fibroblasts with common PI3K inhibitors can result in decreased ECM protein production, supporting this as a viable therapeutic option. Together with the data presented here, this strongly supports a role for PDGF and the PI3K pathways as important drivers of fibrosis in SSc.

Coupling snMultiome with 2 different spatial technologies, one whole transcriptome but low spatial resolution and the other, a 5,000 gene panel at single-cell resolution, allowed us to uncover a distinct profibrotic cellular niche around the vasculature that is enriched in macrophage-fibroblast interactions. These data support the enrichment of immunomodulatory fibroblasts and macrophages in the papillary dermis, the hypodermis, and, most importantly, near the vasculature. This includes a concomitant enrichment of the PI3K/AKT pathway, which indicates activation of these cellular interactions within this site of early pathology in SSc. These spatial transcriptomic analyses indicate regions of early interactions that contribute to disease progression in dermal SSc fibrosis.

This study has limitations, including the relatively small number of patient samples, which limited our statistical power and restricted our ability to compare autoantibody status and patient molecular subtypes.

These findings provide a fully integrated analysis of multiple single-cell and spatial omics approaches and technologies, each on a cohort of untreated patients with early-stage SSc with detailed clinical data. Our study strongly supports the hypothesis that alternatively activated macrophages drive SSc-associated fibroblast activation and provides potentially new context that this is occurring in localized cellular niches around the vasculature. This cellular crosstalk was confirmed at both the transcriptomic and spatial levels, indicating a potential avenue for future treatment targeting either the downstream PI3K/AKT pathway or macrophage-derived PDGF and TGF-β signaling.

## Methods

### Sex as a biological variable.

The distribution of samples collected in this study reflects the real-world overrepresentation of women with SSc: 70% of the samples collected from individuals with SSc were from women, and 30% were from men, and similar findings are reported for both sexes.

### Study design.

The objectives of this study were to identify transcriptional and spatial dynamics of early-stage, untreated SSc skin biopsies. Sample size was determined by a participant recruitment period through a 1-year window, September 2022 to September 2023, for individuals who had early-stage disease (<2 years) and were not receiving drug therapy at the time. Regarding data inclusion and exclusion criteria, all samples from all enrolled patients were included in the analysis. Some samples were excluded from subclustering analysis if fewer than 10 cells of a particular subtype were found for each sample. No outliers were excluded. The research participants were adults (18 to 99 years of age) with active SSc (as per American College of Rheumatology guidelines) and were sampled for sequencing and staining at lesion sites.

### Sample acquisition.

Ten patients with SSc and 4 healthy donors were recruited for snMultiome (10X Genomics) sequencing. The 10 patients with SSc also had matching biopsies to undergo spatial transcriptomic sequencing (10X Genomics’ Visium and Xenium). Skin biopsies were taken from the forearm (lesional, 4 mm punch biopsy) of study participants. This study was approved by the University of Michigan IRB, and all patients gave written consent. The Committee for the Protection of Human Subjects (CPHS) approved all human participant analyses at Dartmouth College’s Geisel School of Medicine.

### snMultiome library preparation, sequencing, and alignment.

Skin biopsies were incubated overnight in 0.4% Dispase (Life Technologies) in HBSS (Gibco) at 4°C. The epidermis and dermis were manually separated. The epidermal layer was digested in 0.25% trypsin-EDTA (Gibco) with 10 U/mL DNase I (Thermo Fisher Scientific) for 1 hour at 37°C, quenched with FBS, and filtered through a 70 μm strainer. The dermis was minced and digested in 0.2% collagenase II (Life Technologies) and 0.2% collagenase V (Sigma-Aldrich) in DMEM for 1.5 hours at 37°C, then filtered through a 70 μm strainer. Cells were counted and then combined at a 1:1 ratio. Using the 10X Genomics Multiome (ATAC + RNA) workflow, single nuclei were isolated and captured on a Chromium X instrument, and Illumina sequencing libraries were prepared following the manufacturer’s protocols (CG000338, CG000365). Libraries were sequenced on an Illumina NovaSeq 6000.

### Cell clustering and cell type annotation.

Read alignment, quality control, gene quantification, and peak assessments for each sample were conducted using 10X Genomics’ Cell Ranger ARC software (v2.0.2) with GRCh38 reference. ATAC and gene expression (GEX) feature-barcode matrices were loaded into Seurat (v4.4.0) and Signac (v1.12.0) for further analyses (46, 47). We included nuclei with greater than 1,000 unique RNA molecules, less than 25,000 total RNA counts, less than 100,000 ATAC fragments, greater than 1,000 ATAC fragments, less than 25% mitochondrial content, a score less than 2 of nucleosome signal measurement, and greater than 2 for transcription start site score. For snRNA-Seq, the raw counts were normalized and scaled using SCTransform, while the remaining mitochondrial content was regressed. The top 2,000 highly variable features were used for principal component analysis, and the top 40 principal components were used for UMAP. Doublets were removed using scDblFinder (v1.14) (48). Harmony (v1.2) was used for the RNA GEX data to correct for batch correction (49). For snATAC-Seq, after merging the objects, we called the consensus peaks using MACS2 (v2.2.9.1) in Signac. The peaks on the nomic blacklist region of GRCh38 were removed. We performed normalization of frequency inverse documented frequency (TF-IDF) using RunTFIDF, identified top features with min.cutoff = 5, and performed latent semantic indexing (LSI) reduction using RunSVD. The top 2:20 dimensions were used for generating the ATAC-based UMAP. We then built a combined weighted nearest neighbor (WNN) graph using FindMultimodalNeighbors with the same 1:40 principal components from the snRNA-Seq data and the 2:20 LSI dimensions from the snATAC-Seq data for joint-UMAP visualization. Our quality filtering resulted in a total of 31,110 single-nuclei data points for combined ATAC and GEX analyses.

Major clusters were identified using FindNeighbors and with FindClusters at a resolution of 0.4 in the WNN UMAP. Cell types were assigned to each cluster using gene expression of cell type–specific markers, as defined by previous publications, in combination with accessible peaks near canonical markers. Given the well-documented cell typing in both GEX and ATAC-Seq data, we could confidently identify major skin cell types using both modalities. A total of 19 clusters were identified, which aligned with 12 total broad cell types. Cell types were also confirmed using the package CAMML to project previously published gene sets of other annotated single-cell transcriptomes (GEO GSE138669) (2). Proportional differences compared between SSc and HC groups using proportional odds regression using the propeller function in the speckle package, enabling standard 2-tailed *t* tests (50).

### Cell type subclustering.

Cell types of interest for this study were subclustered and filtered to determine unique subtypes per disease condition. Multimodal UMAP clustering was performed for each primary cell type cluster, including fibroblasts, immune cells, keratinocytes, and endothelial cells. After initial clustering, an additional filtering step excluded any additional misclassified cell types of interest. SingleR (v2.6) and canonical gene differential expression was used to classify specific cell types when examining immune cell subtypes (51).

### Downstream pathway and ligand-receptor analyses.

To examine sample-level changes in the subclusters, we pseudo-bulked the nuclei transcriptomes by sample and performed GSVA (v1.52.3) on pseudo-bulked samples (52). We also used other downstream pathway analysis methods, such as VAM (v1.1.0), gprofiler2 (v0.2.3), and clusterProfiler (v4.12) (53–55). After subclustering of each primary cell type, we then performed ligand-receptor analysis using CellChat (v2.1.2) to observe potential interactions between subclusters across the entirety of the dataset (56). Interactions were considered significant by Wilcoxon’s rank-sum test comparing the condition and expression of ligands and receptors on the sending and receiving cells: cutoff *P* value 0.05, ligand cumulative logFC greater than 0.05.

### snATAC-Seq transcription factor motif analysis.

Motif enrichment using Signac was completed after finding unique peaks for each cluster or cell type and finding the *cis*-regulatory connections with Cicero (v1.3.9) and monocle3 (v1.3.7) (57, 58). Only peaks with a Cicero score greater than 0.25 were included to accurately find transcription factors in regions that are correlated with gene expression. Per-nuclei motif activity scores were computed using chromVAR (v1.22.1) and USC hg38 genome (59).

### Visium spatial transcriptomic cluster, deconvolution, and downstream analysis.

FFPE histology sections were placed onto Epredia ColorFrost slides, H&E stained on a TissueTek Prisma Stainer (Sakura), and imaged at ×40 magnification on an Aperio GT450 instrument (Leica). Following the Visium CytAssist Spatial Gene Expression workflow (CG000495), tissues were deparaffinized, de-crosslinked, hybridized with the human whole transcriptome probe set (v2), and ligated to form full-length, functional probes. Full-length probes were transferred onto Visium spatially barcoded slides, converted to Illumina sequence libraries, and sequenced on a NextSeq 2000 instrument targeting 50,000 reads per spot. Initial alignment and quality control was done with Space Ranger (v2.0.0). Following initial quality control, Visium samples were integrated and clustered using PRECAST (v1.6.5) and Seurat (v4.4.0) (47, 60). Clusters largely overlapped with the histological architecture of the skin and were appropriately assigned names by their regional function. Assigning clustering nomenclature by functional region was confirmed through cell-type enrichment analysis. Each Visium spot was deconvoluted by cell type, using our previously constructed multiome data, using CARD (v1.1) to find the relative proportion of broad cell types (61). We isolated spots that contained fibroblasts by filtering the spots for greater than 2.5% of the predicted fibroblast composition. Using these isolated spots, we used Variance-Adjusted Mahalanobis (VAM, v1.1.0) to observe the multiome-based subcluster’s enrichment in fibroblast-specific spots (53). Following this procedure, we could also similarly find enrichment of immune cell subclusters, keratinocytes, and gene set enrichment per spot. Using CellChat, we also performed a region-based receptor-ligand interactome analysis between the different regions within each skin section (v2.1.2) (56).

### Microarray analysis.

Preprocessed data were downloaded from GEO GSE56308. Differential expression was performed using limma (v3.60.2). GSVA was performed for the Hallmark gene set, with comparisons done using the Wilcoxon’s rank-sum test. GO term analysis for Molecular Function was performed using ClusterProfiler (v4.12.0).

### Bulk RNA-Seq analysis.

Preprocessed data for Figure 6, D and E, were obtained from Ginkgo Bio (GPDx2 dataset, https://huggingface.co/datasets/ginkgo-datapoints/GDPx2). Control (DMSO-treated) and wortmannin-treated samples were extracted. Sample normalization and differential expression were conducted using DESeq2 (v1.44.0) (62). GSVA was performed for the Hallmark gene set database, with comparisons done using Wilcoxon’s rank-sum tests.

Preprocessed data were downloaded from GEO GSE289407. Sample normalization and differential expression analysis were performed using DESeq2. GSVA was performed for the Reactome database, with comparisons made using the Wilcoxon rank-sum test and 1-way ANOVA.

### Xenium in situ gene expression data analysis.

Matching FFPE histology sections also underwent 10X Genomics’ Xenium in situ gene expression assay. Proprietary iterative in situ hybridization was conducted using the Xenium Prime 5K Human Pan Tissue and Pathways panel. Tissues were sectioned onto Xenium slides and processed according to the manufacturer’s protocol, including the workflow for cell segmentation staining (CG000760). Slides were imaged, cells were segmented, and initial quality control was completed on the Xenium analyzer (v3.0.2.0). Segmented images that had been quality-controlled were then transferred and converted to anndata objects for downstream Python-based analysis methods. Each composite image with gene expression counts underwent quality filtering and spatial clustering analysis methods using SquidPy; cells that contained 100 or more transcripts per cell and genes that were found in 5 or more cells were included (63). Using data previously generated in this study (see *Cell clustering and cell type annotation* above), each cell had a predicted cell-type inferred to batch-specific reference data using Tangram (64). Combined with Tangram predictions, gene expression, and unsupervised clustering, broad cell types were assigned and then subclustered using similar methods. Differential expression was performed in SquidPy by Wilcoxon’s rank-sum test to identify genes enriched in specific cell types and regions. Neighborhood analysis was used to determine cellular niches within the sections. Finally, odds ratio tests were used to identify the likelihood of finding cell types within specific regions.

### Immunofluorescence and immunohistochemical staining.

FFPE tissue sections were cut, baked at 65°C for 30 minutes, deparaffinized, and rehydrated. Antigen retrieval was performed in pH 6 or pH 9 buffer (per manufacturer’s instructions) at 125°C for 30 seconds using a pressure cooker. After cooling, slides were blocked and incubated with primary rabbit antibodies against α-SMA (Abcam, ab5694) and CD163 (MA5-11458), both diluted 1:100. Isotype controls (rabbit IgG, ab172730; mouse IgG1, ab280974) and no-antibody controls were included. Slides were washed (3 times, 5 minutes each, PBST), and then incubated for 30 minutes with secondary antibodies: Alexa Fluor 594 anti-rabbit IgG (711-585-152, Jackson ImmunoResearch) and Alexa Fluor 488 anti-mouse IgG (715-545-151, Jackson ImmunoResearch). After final washes, slides were mounted in ProLong Diamond with DAPI (Invitrogen). Images were acquired using a Zeiss fluorescence microscope. Exposure settings were matched across samples and controls; representative fields are shown.

### Statistics.

Independent biological samples were used for sequencing. Results are described as the mean and were analyzed by Student’s *t* test (unpaired, 2-tailed) or a Wilcoxon’s rank-sum (2-sided) for individual comparisons. Various gene set enrichment tests are used, as indicated in the figure legends and specific sections within the Methods. Significance was set at *P* less than 0.05.

### Study approval.

Before research commenced, IRBs from the University of Michigan (HUM00202522) and Dartmouth College (STUDY00032378) approved the study protocol. The study was conducted per the Declaration of Helsinki and Good Clinical Practice. Written informed consent was obtained prior to the individual’s participation.

### Data availability.

Raw data files for SSc and healthy single-cell multiome and Visium sequencing samples have been deposited in NCBI’s GEO (GSE312129). Raw and preprocessed data for the Xenium spatial transcriptomics data have been deposited in GEO (GSE312932). Supporting data files can be found in Supplemental Tables 1–33.

## Author contributions

HCJ, DK, JEG, and MLW conceptualized the study. HCJ, RP, ZG, and MJM performed analysis. HCJ, XX, LP, RG, LACT, RB, and FK provided experimental resources and assays. HCJ, RP, and ZG conducted the investigation. JEG, FK, PAP, DK, and MLW acquired funding. JEG, DK, FK, and MLW supervised the study. HCJ wrote the original draft. HCJ, RP, PAP, JEG, DK, and MLW reviewed and edited the manuscript. All authors reviewed and approved the final manuscript.

## Funding support

This work is the result of NIH funding, in whole or in part, and is subject to the NIH Public Access Policy. Through acceptance of this federal funding, the NIH has been given a right to make the work publicly available in PubMed Central.

- Department of Defense grants W81XWH-21-1-0878 (to MLW), W81XWH-21-1-0881 (to FWK), and W81XWH-21-1-0880 (to DK).
- National Scleroderma Foundation Pre-doctoral Summer Fellowship (to HCJ and MJM).
- National Institute of Allergy and Infectious Diseases grants R21AI169420 (to PAP and MLW), R21AI178651 (to PAP and MLW), R01AI184620 (to JEG), and R01AI183620 (to JEG and DK).
- National Institute of General Medical Sciences grant P20GM130454 (to MLW).
- National Institute of Arthritis & Musculoskeletal & Skin Disease grant P30AR075043 (to JEG).

## Supplementary Material

Supplemental data

Supporting data values

## Figures and Tables

**Table 1:**
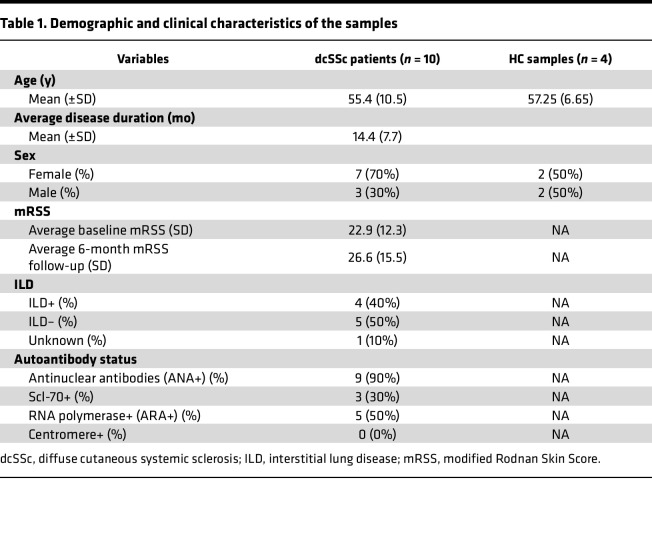
Demographic and clinical characteristics of the samples

## Version 1 12/18/2025

In-Press Preview

## Version 2 02/09/2026

Electronic publication
