# Supplementary material for: Multimodal analyses of early, untreated systemic sclerosis skin identify a proinflammatory vascular niche of macrophage-fibroblast signaling
Source: JCI Insight. 2025 Dec 18;11(3):e198954. doi: 10.1172/jci.insight.198954 (PMC12893110; doi:10.1172/jci.insight.198954)
Supplement: Supplemental data [file jciinsight-11-198954-s203.pdf]

Supplementary Figures

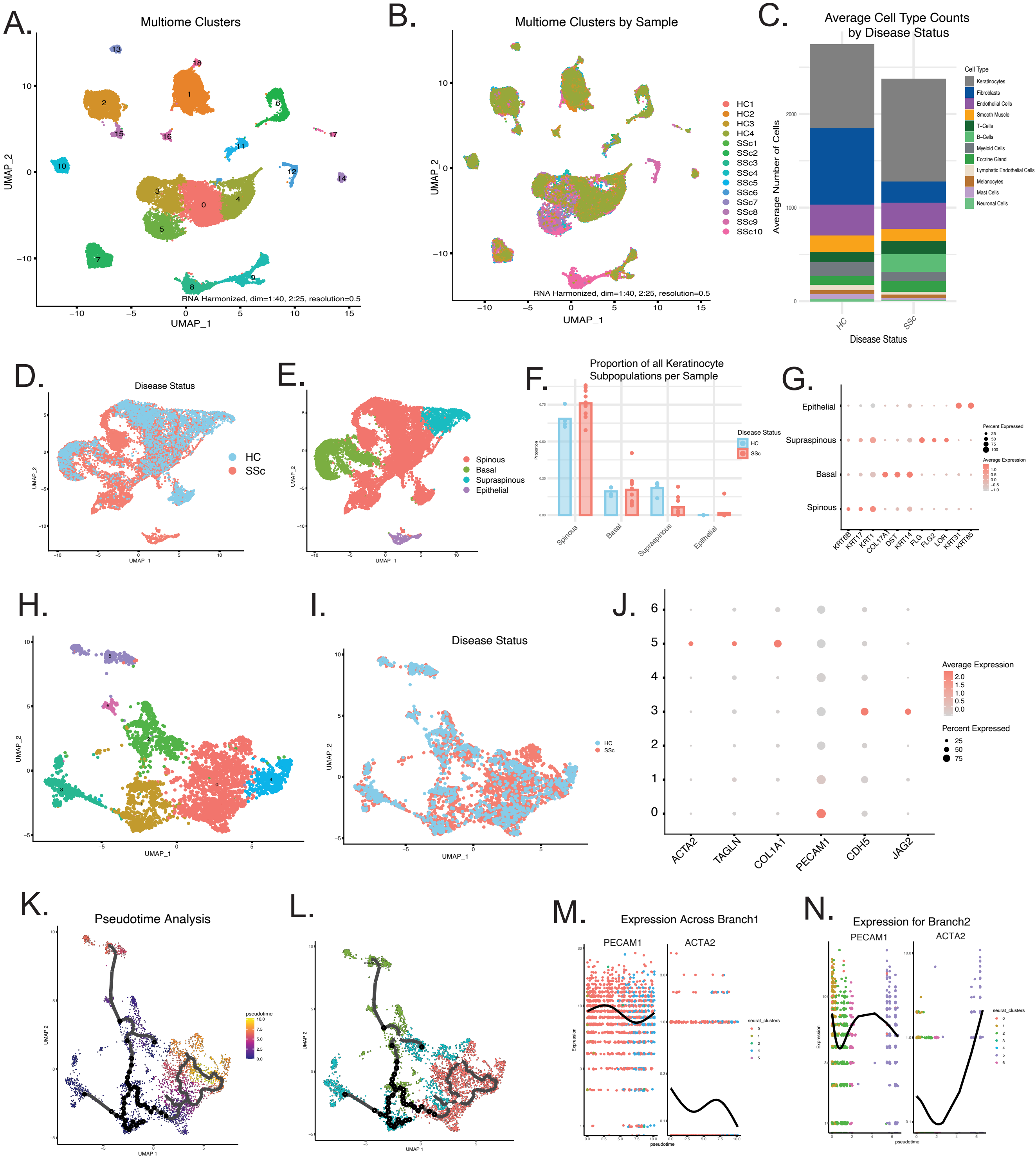

Supplementary Figure 1: Multiome unsupervised clustering. A) Nuclei clustered and colored by unsupervised labels for initial cell type assessment. Data were harmonized for batch correction. Dimensions for RNA and ATAC data. B) Nuclei colored by sample origin name, demonstrating even distribution of cell types by original sample ID. C) Total nuclei counts differences by diseases for all cell types. D) UMAP of keratinocyte subclustering colored by disease status. E) UMAP of keratinocyte subpopulations named through canonical subtype. F) Proportion of each keratinocyte subpopulation to all keratinocytes shows no significant difference in canonical subclusters by disease. G) Differentially expressed canonical markers (top 2) for each keratinocyte subtype. H) UMAP of endothelial subclusters. I) UMAP of endothelial cell subclustering colored by disease status. J) Expression patterns of profibrotic markers (ACTA2, TAGLN, COL1A1) and classical endothelial cell markers (PECAM1, CDH5, JAG2) across all endothelial cell clusters. K) Pseudotime analysis with assigned root node, circled in green, across the endothelial cell subclusters, indicating divergent branches of trajectory. L) Branches identified to separate out potential endothelial cell lineages. M) Expression of PECAM1 and ACTA2 across the pseudotime space of Branch1. N) Expression of PECAM1 and ACTA2 across the pseudotime space of Branch2.

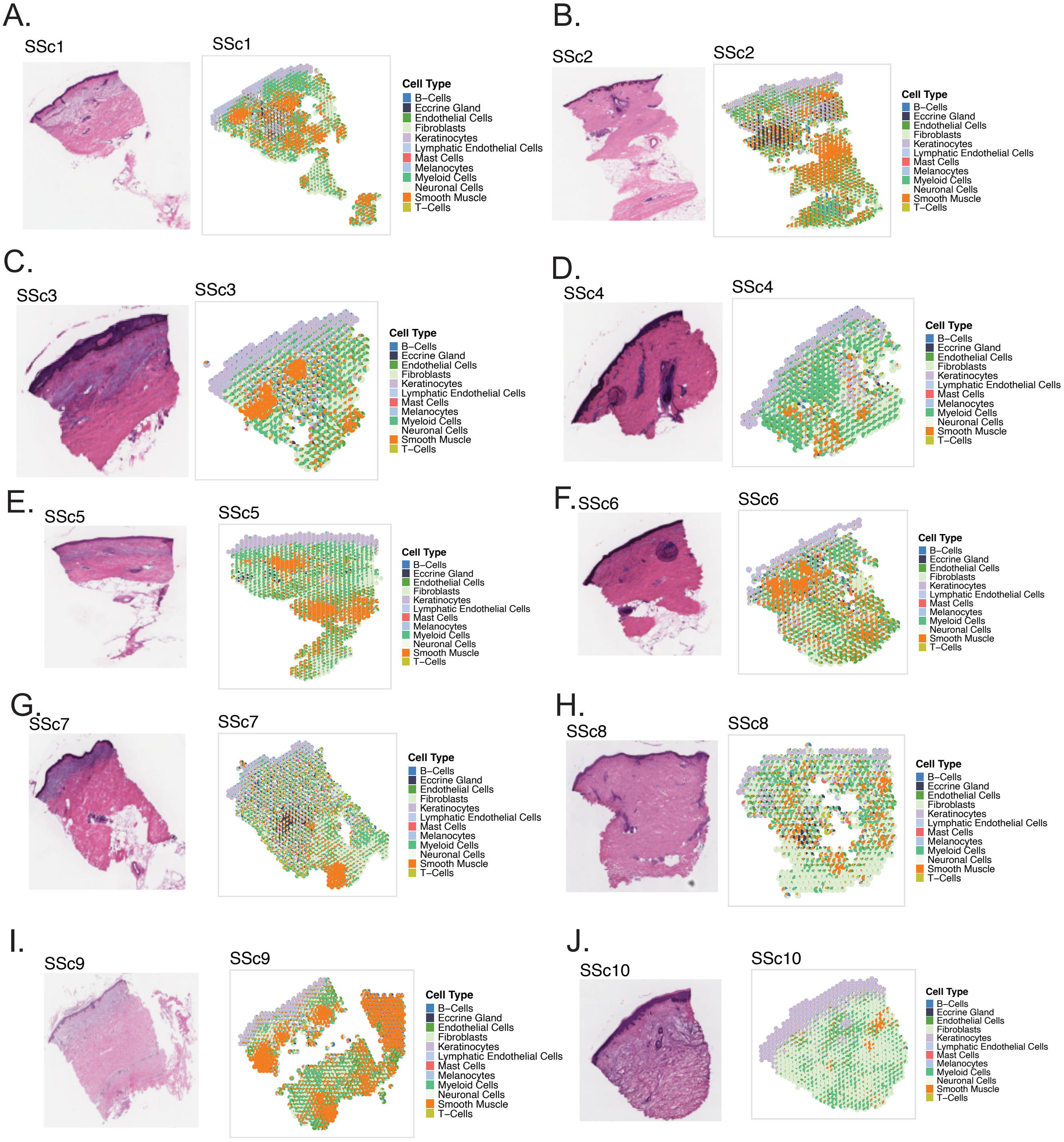

A.
SSc1
SSc1
Cell Type
B–Cells
Eccrine Gland
Endothelial Cells
Fibroblasts
Keratinocytes
Lymphatic Endothelial Cells
Mast Cells
Melanocytes
Myeloid Cells
Neuronal Cells
Smooth Muscle
T–Cells
B.
SSc2
SSc2
Cell Type
B–Cells
Eccrine Gland
Endothelial Cells
Fibroblasts
Keratinocytes
Lymphatic Endothelial Cells
Mast Cells
Melanocytes
Myeloid Cells
Neuronal Cells
Smooth Muscle
T–Cells
C.
SSc3
SSc3
Cell Type
B–Cells
Eccrine Gland
Endothelial Cells
Fibroblasts
Keratinocytes
Lymphatic Endothelial Cells
Mast Cells
Melanocytes
Myeloid Cells
Neuronal Cells
Smooth Muscle
T–Cells
D.
SSc4
SSc4
Cell Type
B–Cells
Eccrine Gland
Endothelial Cells
Fibroblasts
Keratinocytes
Lymphatic Endothelial Cells
Mast Cells
Melanocytes
Myeloid Cells
Neuronal Cells
Smooth Muscle
T–Cells
E.
SSc5
SSc5
Cell Type
B–Cells
Eccrine Gland
Endothelial Cells
Fibroblasts
Keratinocytes
Lymphatic Endothelial Cells
Mast Cells
Melanocytes
Myeloid Cells
Neuronal Cells
Smooth Muscle
T–Cells
F.
SSc6
SSc6
Cell Type
B–Cells
Eccrine Gland
Endothelial Cells
Fibroblasts
Keratinocytes
Lymphatic Endothelial Cells
Mast Cells
Melanocytes
Myeloid Cells
Neuronal Cells
Smooth Muscle
T–Cells
G.
SSc7
SSc7
Cell Type
B–Cells
Eccrine Gland
Endothelial Cells
Fibroblasts
Keratinocytes
Lymphatic Endothelial Cells
Mast Cells
Melanocytes
Myeloid Cells
Neuronal Cells
Smooth Muscle
T–Cells
H.
SSc8
SSc8
Cell Type
B–Cells
Eccrine Gland
Endothelial Cells
Fibroblasts
Keratinocytes
Lymphatic Endothelial Cells
Mast Cells
Melanocytes
Myeloid Cells
Neuronal Cells
Smooth Muscle
T–Cells
I.
SSc9
SSc9
Cell Type
B–Cells
Eccrine Gland
Endothelial Cells
Fibroblasts
Keratinocytes
Lymphatic Endothelial Cells
Mast Cells
Melanocytes
Myeloid Cells
Neuronal Cells
Smooth Muscle
T–Cells
J.
SSc10
SSc10
Cell Type
B–Cells
Eccrine Gland
Endothelial Cells
Fibroblasts
Keratinocytes
Lymphatic Endothelial Cells
Mast Cells
Melanocytes
Myeloid Cells
Neuronal Cells
Smooth Muscle
T–Cells

Supplementary Figure 2: Spot Deconvolution for each sample and histology section. A-J) Pie chart plots for each spot with estimated cell-type proportions computed with CARD using the Multiome data as a reference. Underlying H&E stains for each tissue section sequenced.

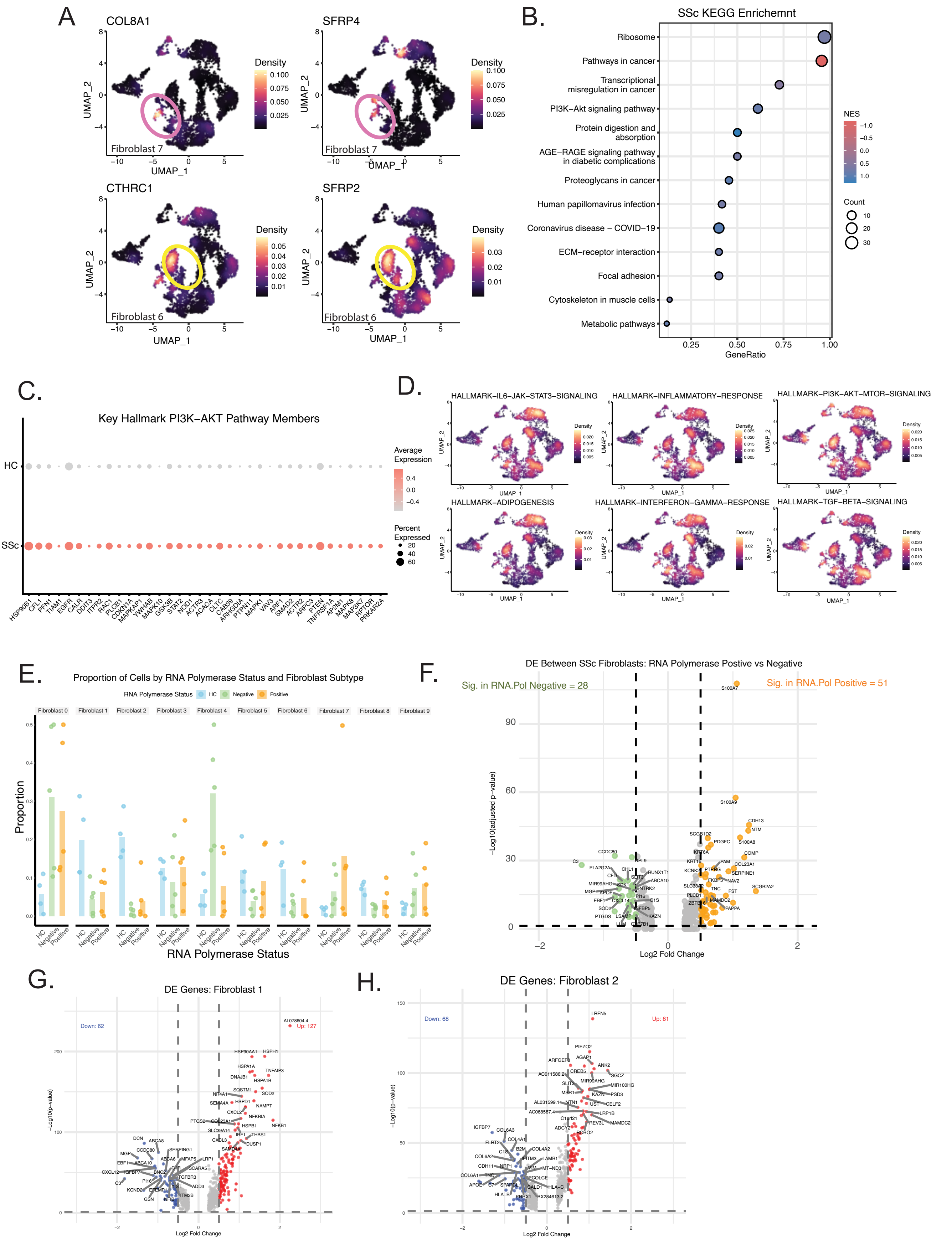
A.
COL8A1
SFRP4
CTHRC1
SFRP2
Fibroblast 7
Fibroblast 6
Density
UMAP_1
UMAP_2
B.
SSc KEGG Enrichemnt
Ribosome
Pathways in cancer
Transcriptional misregulation in cancer
PI3K–Akt signaling pathway
Protein digestion and absorption
AGE–RAGE signaling pathway in diabetic complications
Proteoglycans in cancer
Human papillomavirus infection
Coronavirus disease – COVID–19
ECM–receptor interaction
Focal adhesion
Cytoskeleton in muscle cells
Metabolic pathways
GeneRatio
NES
Count
C.
Key Hallmark PI3K–AKT Pathway Members
HC
SSc
Average Expression
Percent Expressed
D.
HALLMARK–IL6–JAK–STAT3–SIGNALING
HALLMARK–INFLAMMATORY–RESPONSE
HALLMARK–PI3K–AKT–MTOR–SIGNALING
HALLMARK–ADIPOGENESIS
HALLMARK–INTERFERON–GAMMA–RESPONSE
HALLMARK–TGF–BETA–SIGNALING
E.
Proportion of Cells by RNA Polymerase Status and Fibroblast Subtype
RNA Polymerase Status
HC
Negative
Positive
Proportion
RNA Polymerase Status
F.
DE Between SSc Fibroblasts: RNA Polymerase Postive vs Negative
Sig. in RNA.Pol Negative = 28
Sig. in RNA.Pol Positive = 51
–Log10(adjusted p–value)
Log2 Fold Change
G.
DE Genes: Fibroblast 1
Down: 62
Up: 127
–Log10(p–value)
Log2 Fold Change
H.
DE Genes: Fibroblast 2
Down: 68
Up: 81
–Log10(p–value)
Log2 Fold Change

Supplementary Figure 3: Fibroblast Subcluster and pathway interrogation. A) Density plots of common canonical markers of fibroblast subclusters. COL8A1, SFRP4, CTHRC1, and SFRP4 are enriched in Fibroblast 7 and Fibroblast 6. B) GO term analysis, using clusterProfiler, demonstrates enrichment of key KEGG pathways in SSc fibroblasts compared to HC. C) Hallmark geneset PI3K-AKT-MTOR pathway genes differentially expressed between SSc and HC fibroblasts. D) VAM enrichment of key Hallmark genesets across the fibroblast subclusters. E) Proportional comparison of fibroblast subclusters by RNA polymerase autoantibody status. F) Volcano plot of differentially expressed genes of all fibroblasts from SSc samples compared by RNA polymerase status. G) Volcano plot of differential expression results of Fibroblast 1 cluster compared to all other Fibroblast clusters. H) Volcano plot of differential expression results of Fibroblast 2 cluster compared to all other Fibroblast clusters.

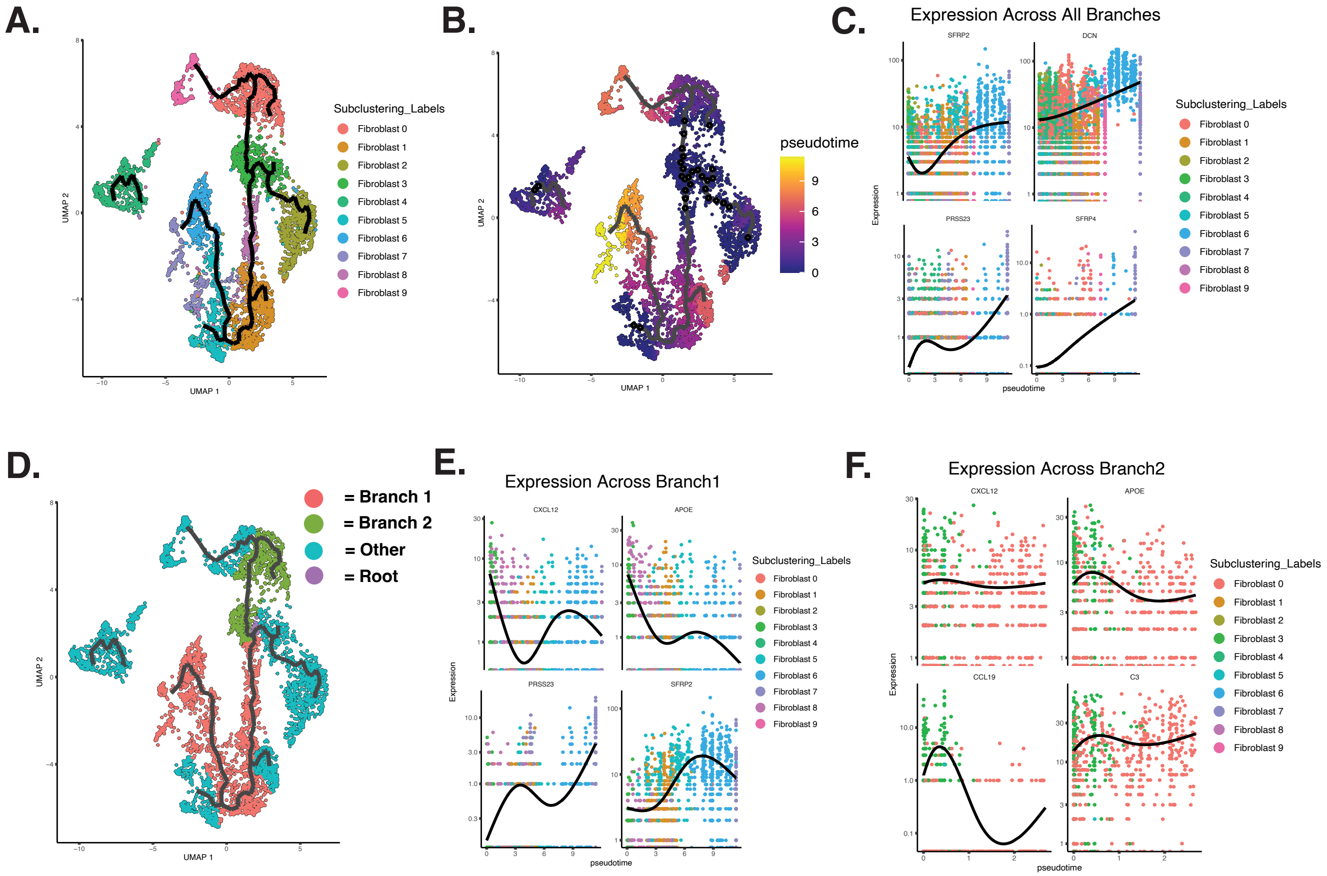
A.
B.
C.
Expression Across All Branches
D.
E.
Expression Across Branch1
F.
Expression Across Branch2
Subclustering_Labels
Fibroblast 0
Fibroblast 1
Fibroblast 2
Fibroblast 3
Fibroblast 4
Fibroblast 5
Fibroblast 6
Fibroblast 7
Fibroblast 8
Fibroblast 9
pseudotime
= Branch 1
= Branch 2
= Other
= Root
UMAP 1
UMAP 2
Expression
SFRP2
DCN
PRSS23
SFRP4
CXCL12
APOE
CCL19
C3

Supplemental Figure 4: Fibroblast pseudotime analysis shows divergent trajectories. A) Pseudotime path with overlaid with fibroblast subclusters. Fibroblast 3 set as root node given central and basal expression patterns. B) Pseudotime path, with cells colored by resultant pseudotime. Fibroblast 7 and 9 are at the end of the total trajectory path when Fibroblast 3 is set as the root node. C) Gene expression plotted across all pseudotime branches shows an increase in fibrotic markers at the tail end of the pseudotime graph. D) UMAP with pseudotime path and cells marked by key pseudotime branches. E) Key genes expressed across Branch 1. F) Key genes expressed across Branch 2.

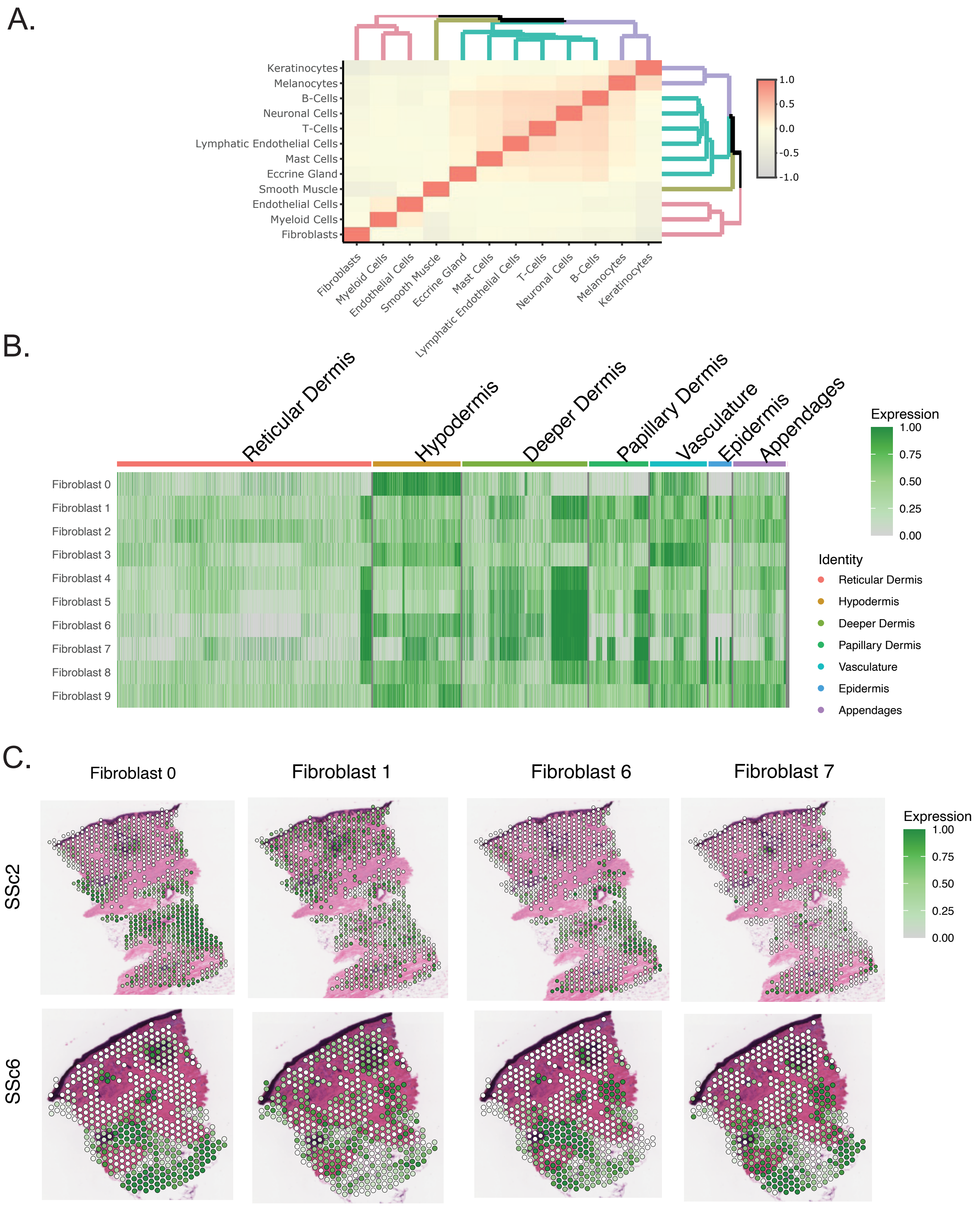

A.
Keratinocytes
Melanocytes
B-Cells
Neuronal Cells
T-Cells
Lymphatic Endothelial Cells
Mast Cells
Eccrine Gland
Smooth Muscle
Endothelial Cells
Myeloid Cells
Fibroblasts
1.0
0.5
0.0
-0.5
-1.0
B.
Reticular Dermis
Hypodermis
Deeper Dermis
Papillary Dermis
Vasculature
Epidermis
Appendages
Fibroblast 0
Fibroblast 1
Fibroblast 2
Fibroblast 3
Fibroblast 4
Fibroblast 5
Fibroblast 6
Fibroblast 7
Fibroblast 8
Fibroblast 9
Expression
1.00
0.75
0.50
0.25
0.00
Identity
C.
Fibroblast 0
Fibroblast 1
Fibroblast 6
Fibroblast 7
SSc2
SSc6

Supplementary Figure 5: Spatial distribution of fibroblast subclusters. A) Correlation of deconvoluted cell types found within the same spots across the all the tissues sections. The most likely cell types to compose spots with fibroblasts, other than other fibroblasts, are endothelial cells or myeloid cells. B) VAM enrichment scores of fibroblast subcluster (in fibroblast containing spots only) genesets, based on differential expression per cluster, across the spatial landscape of two representative sections. C) Heatmap showing VAM enrichment scores for fibroblast subcluster genesets within each region.

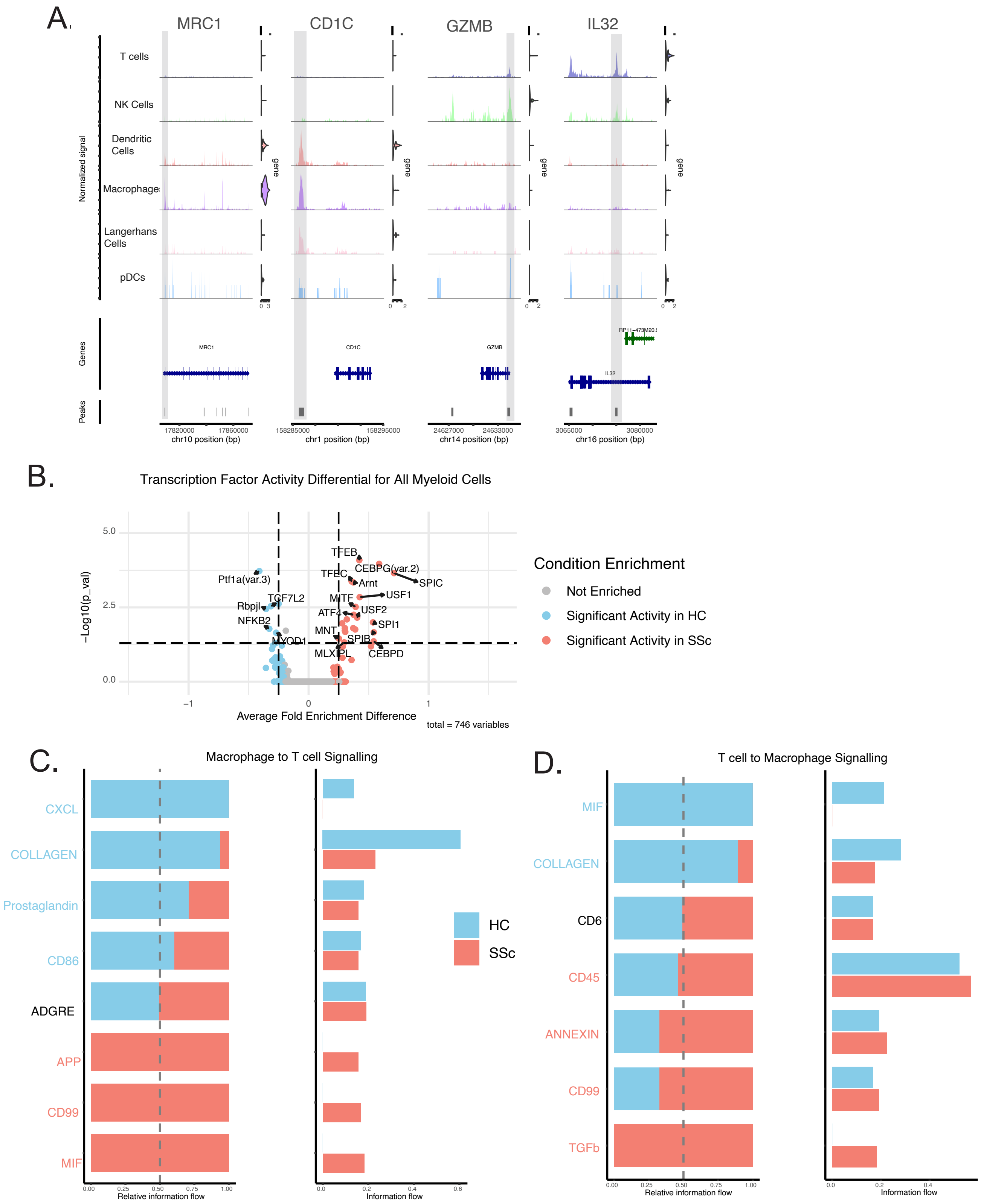
A.
MRC1
CD1C
GZMB
IL32
Normalized signal
T cells
NK Cells
Dendritic Cells
Macrophage
Langerhans Cells
pDCs
gene
Genes
Peaks
chr10 position (bp)
chr1 position (bp)
chr14 position (bp)
chr16 position (bp)
B.
Transcription Factor Activity Differential for All Myeloid Cells
−Log10(p_val)
Average Fold Enrichment Difference
total = 746 variables
Condition Enrichment
Not Enriched
Significant Activity in HC
Significant Activity in SSc
TFEB
CEBPG(var.2)
TFEC
Arnt
SPIC
Ptf1a(var.3)
USF1
TCF7L2
MITF
Rbpjl
USF2
ATF4
NFKB2
SPI1
MNT
SPIB
MYOD1
MLXIPL
CEBPD
C.
Macrophage to T cell Signalling
CXCL
COLLAGEN
Prostaglandin
CD86
ADGRE
APP
CD99
MIF
Relative information flow
Information flow
HC
SSc
D.
T cell to Macrophage Signalling
MIF
COLLAGEN
CD6
CD45
ANNEXIN
CD99
TGFb
Relative information flow
Information flow

Supplementary Figure 6: Key immune cell subtypes confirmed with ATAC-seq data. A) Immune cell subtypes identified by differentially accessible chromatin regions near canonical genes of interest. Peak before MRC1 is differentially accessible in the macrophages, with matching MRC1 gene expression (right). Peaks near CD1C, GZMB, and IL32 in Dendritic Cells, NK cells, and T-cells. B) Volcano plot of transcription factor enrichment by inferred activity in myeloid cells compared by disease status. C) Ligand-receptor results of macrophage to T cell inferred signaling on a relative and absolute information flow of ligand-receptor pathways. Significant pathways are colored, while pathways not found to be statistically significant compared by disease status. D) Ligand-receptor results of T cell to macrophage inferred signaling on a relative and absolute information flow of ligand-receptor pathways.

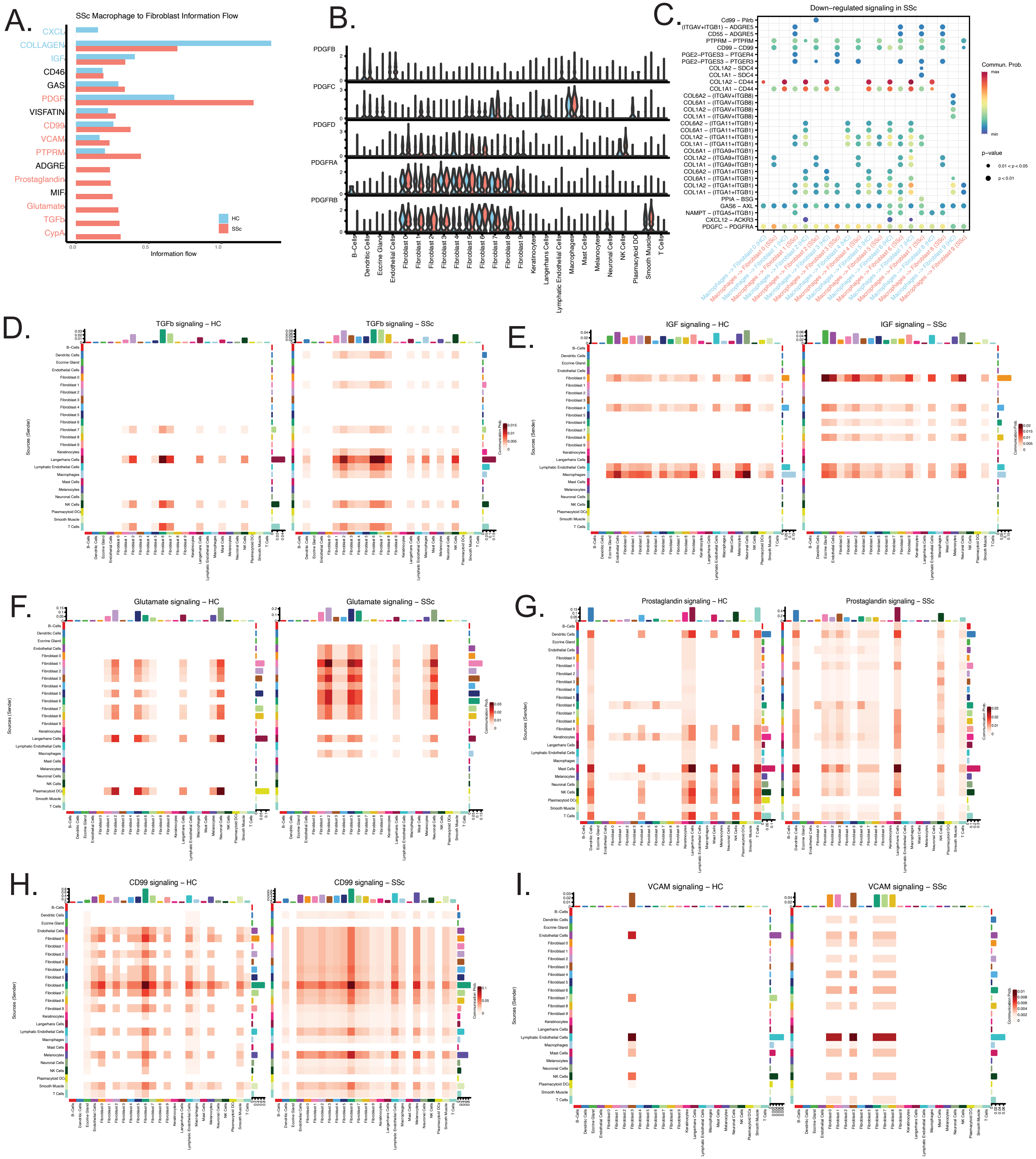
A.
SSc Macrophage to Fibroblast Information Flow
CXCL
COLLAGEN
IGF
CD46
GAS
PDGF
VISFATIN
CD99
VCAM
PTPRM
ADGRE
Prostaglandin
MIF
Glutamate
TGFb
CypA
HC
SSc
0.0
0.5
1.0
Information flow
B.
PDGFB
PDGFC
PDGFD
PDGFRA
PDGFRB
B−Cells
Dendritic Cells
Eccrine Gland
Endothelial Cells
Fibroblast 0
Fibroblast 1
Fibroblast 2
Fibroblast 3
Fibroblast 4
Fibroblast 5
Fibroblast 6
Fibroblast 7
Fibroblast 8
Fibroblast 9
Keratinocytes
Langerhans Cells
Lymphatic Endothelial Cells
Macrophages
Mast Cells
Melanocytes
Neuronal Cells
NK Cells
Plasmacytoid DC
Smooth Muscle
T Cells
C.
Down−regulated signaling in SSc
Cd99 − Pilrb
(ITGAV+ITGB1) − ADGRE5
CD55 − ADGRE5
PTPRM − PTPRM
CD99 − CD99
PGE2−PTGES3 − PTGER4
PGE2−PTGES3 − PTGER3
COL1A2 − SDC4
COL1A1 − SDC4
COL1A2 − CD44
COL1A1 − CD44
COL6A2 − (ITGAV+ITGB8)
COL6A1 − (ITGAV+ITGB8)
COL1A2 − (ITGAV+ITGB8)
COL1A1 − (ITGAV+ITGB8)
COL6A2 − (ITGA11+ITGB1)
COL6A1 − (ITGA11+ITGB1)
COL1A2 − (ITGA11+ITGB1)
COL1A1 − (ITGA11+ITGB1)
COL6A1 − (ITGA9+ITGB1)
COL1A2 − (ITGA9+ITGB1)
COL1A1 − (ITGA9+ITGB1)
COL6A2 − (ITGA1+ITGB1)
COL6A1 − (ITGA1+ITGB1)
COL1A2 − (ITGA1+ITGB1)
COL1A1 − (ITGA1+ITGB1)
PPIA − BSG
GAS6 − AXL
NAMPT − (ITGA5+ITGB1)
CXCL12 − ACKR3
PDGFC − PDGFRA
Commun. Prob.
max
min
p−value
0.01 < p < 0.05
p < 0.01
D.
TGFb signaling − HC
TGFb signaling − SSc
Sources (Sender)
Communication Prob.
E.
IGF signaling − HC
IGF signaling − SSc
F.
Glutamate signaling − HC
Glutamate signaling − SSc
G.
Prostaglandin signaling − HC
Prostaglandin signaling − SSc
H.
CD99 signaling − HC
CD99 signaling − SSc
I.
VCAM signaling − HC
VCAM signaling − SSc

Supplementary Figure 7: Expanded Ligand-Receptor Analyses. A) Sum of predicted interactions between macrophages and fibroblasts comparing SSc and HC (non-relative). B) Expression of PDGF ligands and receptors included in pathway prediction by all cell types comparing between disease state. C) Most downregulated signaling interactions are by exactly the ligand-receptor pair between macrophages and fibroblasts. Filtered by $p < 0.05$, tested by Wilcoxon rank sum test. D) TGFb signaling interaction probability in all cell types compared by disease state. E) IGF signaling interaction probability in all cell types compared by disease state. F) Glutamate signaling interaction probability in all cell types compared by disease state. G) Prostaglandin signaling interaction probability in all cell types compared by disease state. H) CD99 signaling interaction probability in all cell types compared by disease state. I) VCAM signaling interaction probability in all cell types compared by disease state.

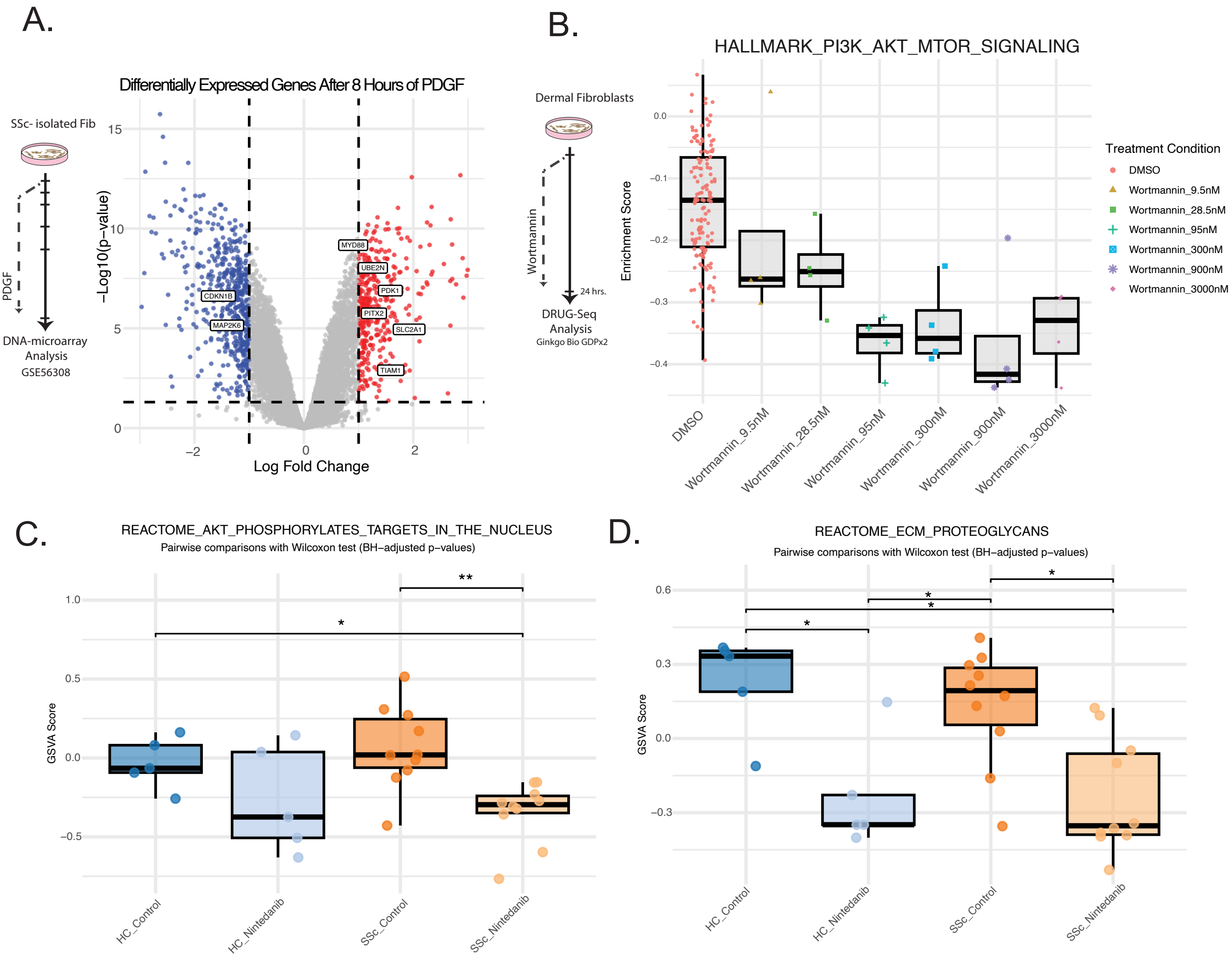
A.
Differentially Expressed Genes After 8 Hours of PDGF
SSc- isolated Fib
PDGF
DNA-microarray Analysis
GSE56308
−Log10(p−value)
Log Fold Change
CDKN1B
MAP2K6
MYD88
UBE2N
PDK1
PITX2
SLC2A1
TIAM1
B.
HALLMARK_PI3K_AKT_MTOR_SIGNALING
Dermal Fibroblasts
Wortmannin
24 hrs.
DRUG-Seq Analysis
Ginkgo Bio GDPx2
Enrichment Score
Treatment Condition
DMSO
Wortmannin_9.5nM
Wortmannin_28.5nM
Wortmannin_95nM
Wortmannin_300nM
Wortmannin_900nM
Wortmannin_3000nM
C.
REACTOME_AKT_PHOSPHORYLATES_TARGETS_IN_THE_NUCLEUS
Pairwise comparisons with Wilcoxon test (BH−adjusted p−values)
GSVA Score
HC_Control
HC_Nintedanib
SSc_Control
SSc_Nintedanib
D.
REACTOME_ECM_PROTEOGLYCANS
Pairwise comparisons with Wilcoxon test (BH−adjusted p−values)
GSVA Score
HC_Control
HC_Nintedanib
SSc_Control
SSc_Nintedanib

Supplementary Figure 8: Bulk gene expression analyses. A) Volcano plot with differentially expressed genes comparing eight hours of PDGF exposure to baseline. Differentially expressed PI3K-AKT pathway genes are highlighted. B) Gene set enrichment comparison of DMSO treated fibroblasts or fibroblasts treated with different concentrations of Wortmannin. The Hallmark PI3K-AKT-MTOR pathway is significantly reduced in fibroblasts treated with 28.5 nM and higher concentrations. C) Box plot of GSVA scores for Reactome database's ECM proteoglycans pathway, showing increased expression in untreated 3D tissues with significant reduction upon Nintedanib treatment. D) GSVA scores of Reactome's AKT phosphorylation targets with an SSc-specific response to Nintedanib treatment.

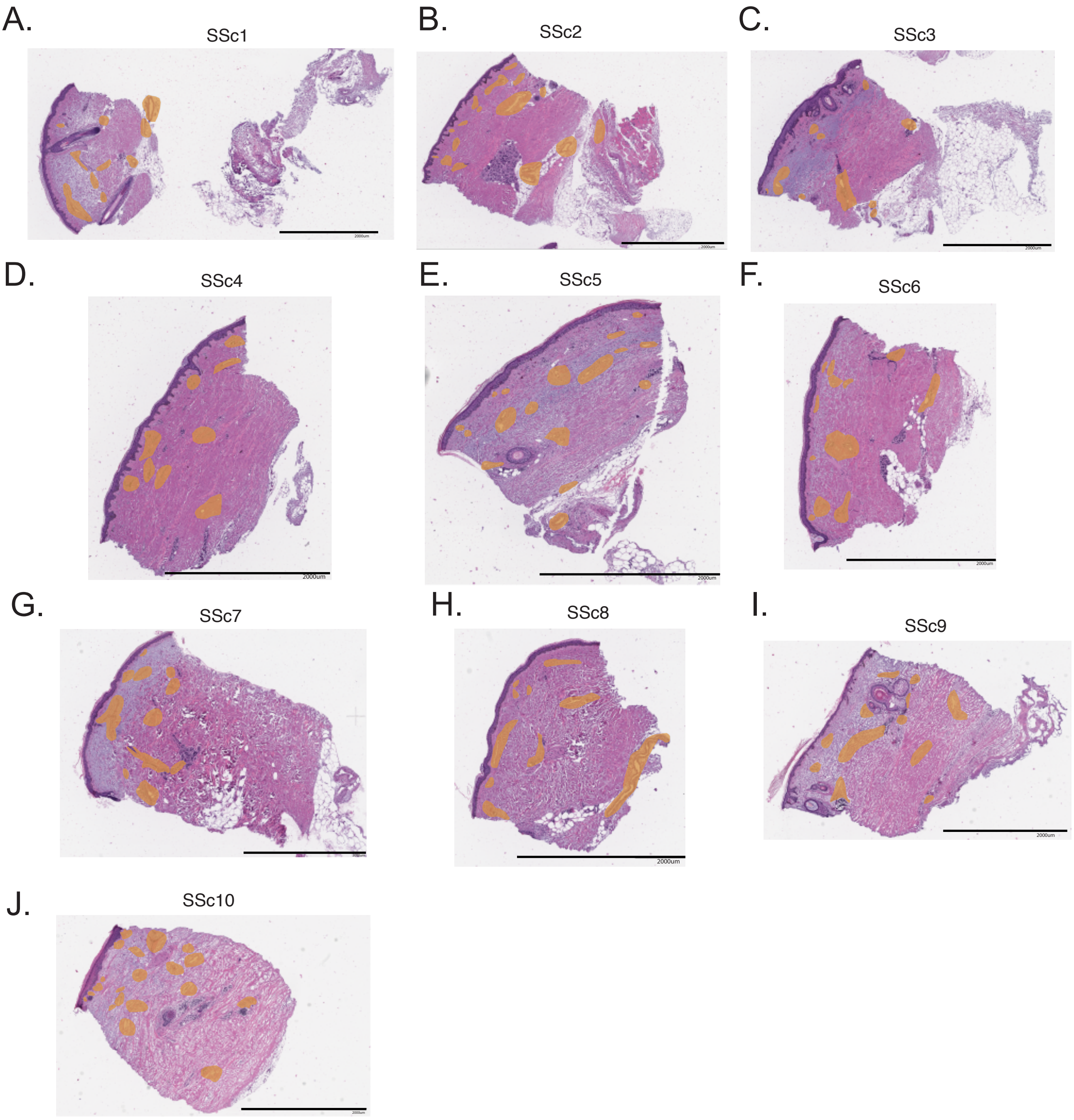
A.
SSc1
B.
SSc2
C.
SSc3
D.
SSc4
2000um
E.
SSc5
F.
SSc6
G.
SSc7
H.
SSc8
2000um
I.
SSc9
J.
SSc10

Supplementary Figure 9: Vasculature regions for each tissue section. A-J) Each tissue section used for Xenium assay (stained with H&E) overlayed with manually identified vasculature regions in orange. Bar indicates 2000 um.

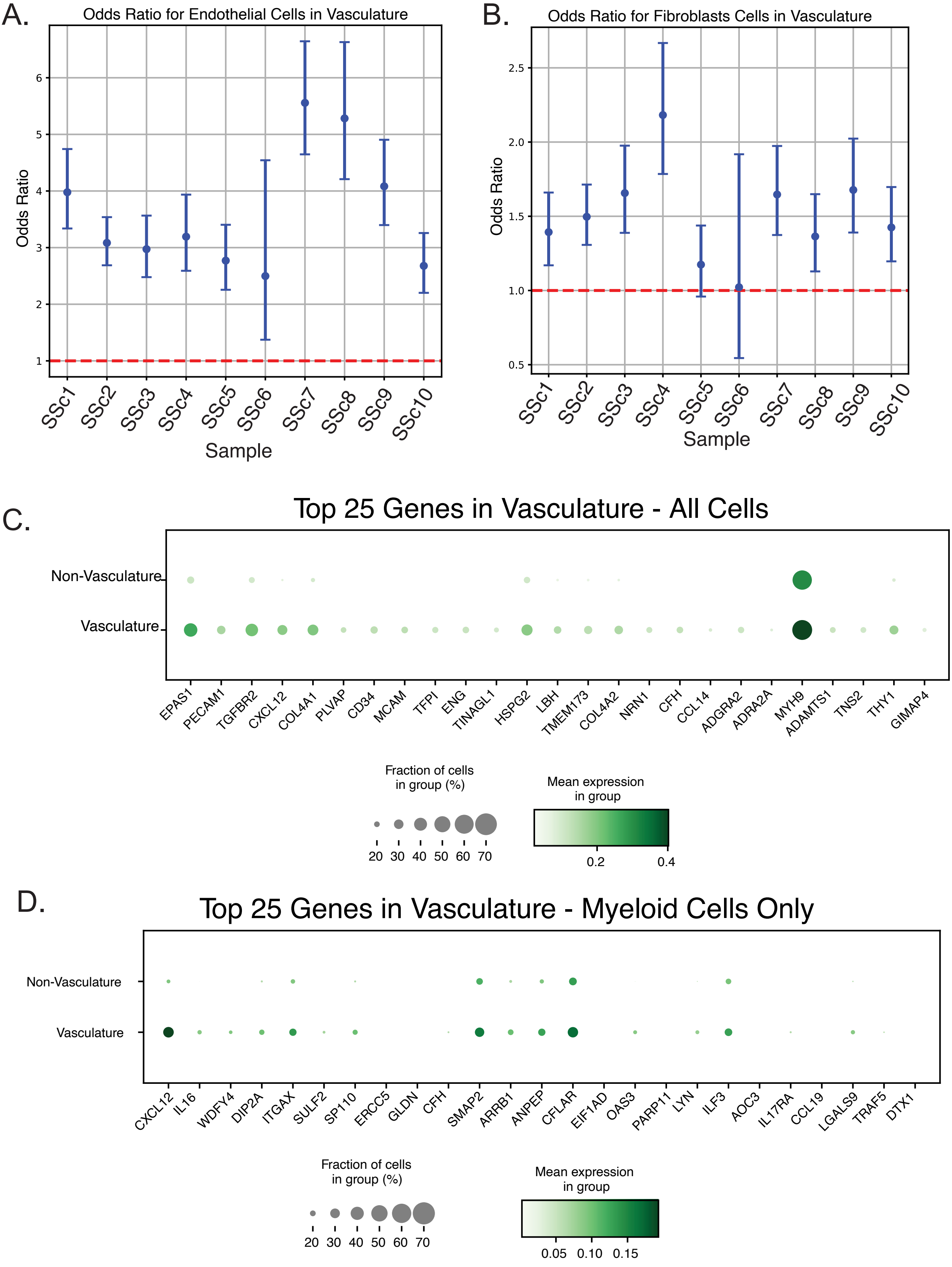
A.
Odds Ratio for Endothelial Cells in Vasculature
Odds Ratio
Sample
SSc1 SSc2 SSc3 SSc4 SSc5 SSc6 SSc7 SSc8 SSc9 SSc10
1 2 3 4 5 6
B.
Odds Ratio for Fibroblasts Cells in Vasculature
Odds Ratio
Sample
SSc1 SSc2 SSc3 SSc4 SSc5 SSc6 SSc7 SSc8 SSc9 SSc10
0.5 1.0 1.5 2.0 2.5
C.
Top 25 Genes in Vasculature - All Cells
Non-Vasculature
Vasculature
EPAS1 PECAM1 TGFBR2 CXCL12 COL4A1 PLVAP CD34 MCAM TFPI ENG TINAGL1 HSPG2 LBH TMEM173 COL4A2 NRN1 CFH CCL14 ADGRA2 ADRA2A MYH9 ADAMTS1 TNS2 THY1 GIMAP4
Fraction of cells in group (%)
20 30 40 50 60 70
Mean expression in group
0.2 0.4
D.
Top 25 Genes in Vasculature - Myeloid Cells Only
Non-Vasculature
Vasculature
CXCL12 IL16 WDFY4 DIP2A ITGAX SULF2 SP110 ERCC5 GLDN CFH SMAP2 ARRB1 ANPEP CFLAR EIF1AD OAS3 PARP11 LYN ILF3 AOC3 IL17RA CCL19 LGALS9 TRAF5 DTX1
Fraction of cells in group (%)
20 30 40 50 60 70
Mean expression in group
0.05 0.10 0.15

Supplementary Figure 10: Xenium results show the specificity of vasculature regions. A) Odds ratio results show a significant probability of finding endothelial cells in the vasculature regions in all 10 Xenim sections. B) Fibroblasts are often found near vasculature regions as confirmed by odds ratio test, but are often dispersed across the tissue sections. C) 25 differentially expressed genes of fibroblasts in the vasculature region (Wilcoxon Rank Sum Test, sorted by LogFC, all genes $p < 0.05$). D) 25 differentially expressed genes of myeloid cells only in the vasculature region (Wilcoxon Rank Sum Test, sorted by LogFC, all genes $p < 0.05$).

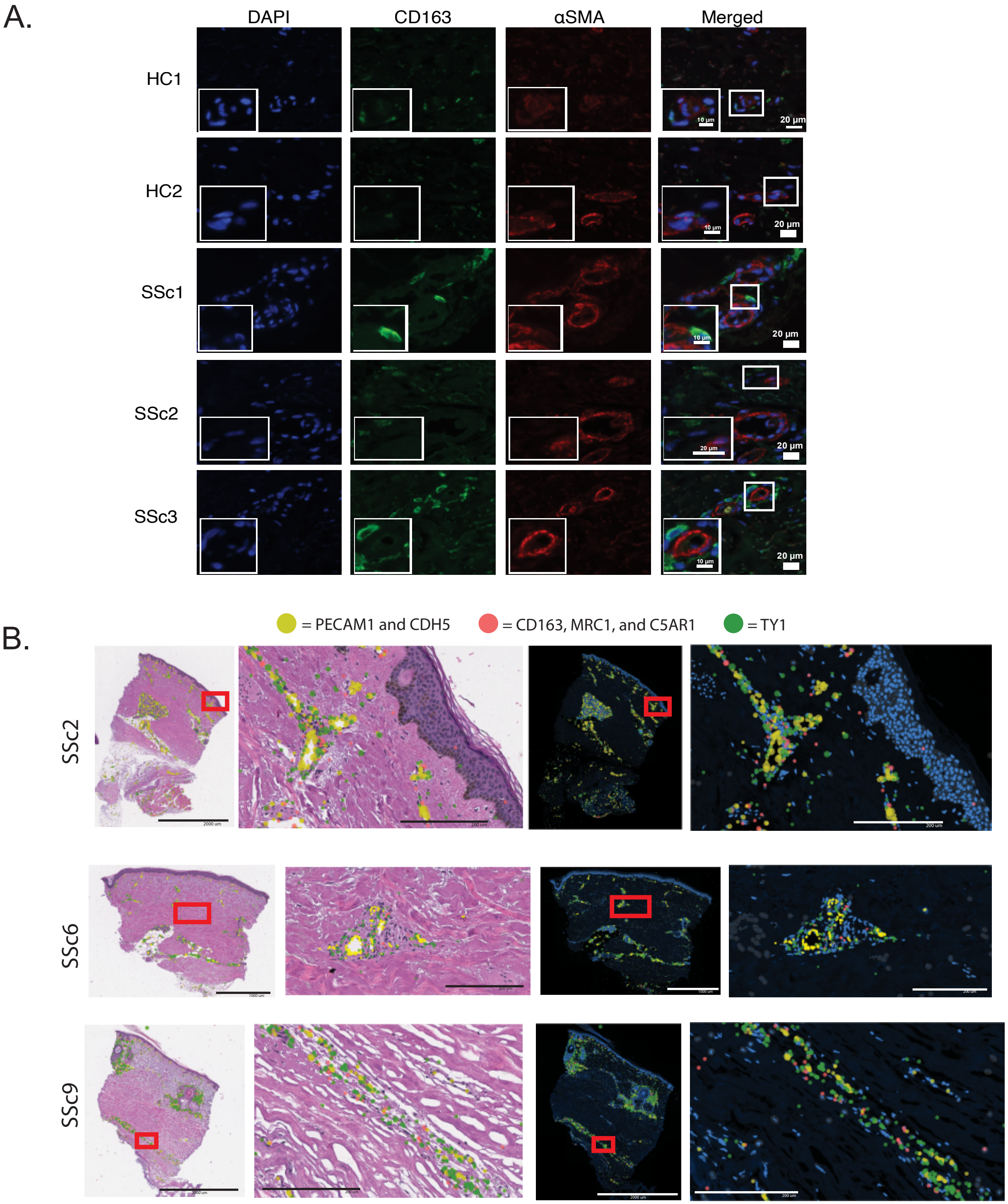
A.
DAPI
CD163
αSMA
Merged
HC1
HC2
SSc1
SSc2
SSc3
10 μm
20 μm
B.
= PECAM1 and CDH5
= CD163, MRC1, and C5AR1
= TY1
SSc2
SSc6
SSc9

Supplementary Figure 11: Immunofluorescence staining of CD163-positive cells and aSMA-positive cells. A) All samples included in the dataset include 2 HCs and 3 SSc samples. The size bar represents 20 µm. Inset shows the outlined region, the inset size bar represents 10 µm. B) Xenium overlay results of selected sections. *PECAM* and *CDH5* molecules are pseudocolored yellow to identify endothelial cells, *CD163*, *MRC1*, and *C5AR1* are combined and pseudocolored red to identify macrophages, and *THY1* molecules are pseudocolored green to identify fibroblasts. H&E overview and inlay are displayed alongside DAPI (blue) counterstain overlay and inlay of the same regions. Measurement bars indicated 2000 um and 200 um in the overview and inlay, respectively.
